# Uncertainty and variability in models of the cardiac action potential: Can we build trustworthy models?

**DOI:** 10.1016/j.yjmcc.2015.11.018

**Published:** 2016-07

**Authors:** Ross H. Johnstone, Eugene T.Y. Chang, Rémi Bardenet, Teun P. de Boer, David J. Gavaghan, Pras Pathmanathan, Richard H. Clayton, Gary R. Mirams

**Affiliations:** a Computational Biology, Dept. of Computer Science, University of Oxford, Oxford OX1 3QD, UK; b Insigneo Institute for in-silico Medicine and Department of Computer Science, University of Sheffield, Sheffield S1 4DP, UK; c CNRS & CRIStAL, Université de Lille, 59651 Villeneuve d'Ascq, France; d Division of Heart & Lungs, Department of Medical Physiology, University Medical Center Utrecht, Utrecht, The Netherlands; e U.S. Food and Drug Administration, 10903 New Hampshire Avenue, Silver Spring, MD 20993, USA

**Keywords:** AP[D], Action Potential [Duration], CMA–ES, Covariance Matrix Adaptation–Evolution Strategy, GP, Gaussian Process, MCMC, Markov Chain Monte Carlo, NLME, Non-Linear Mixed Effects, TP06, the ten Tusscher *et al.* (2006) [45] action potential model, UQ, Uncertainty Quantification, V_m_, trans-membrane Voltage, VVUQ, Verification, Validation & Uncertainty Quantification, Uncertainty quantification, Cardiac electrophysiology, Mathematical model, Probability

## Abstract

Cardiac electrophysiology models have been developed for over 50 years, and now include detailed descriptions of individual ion currents and sub-cellular calcium handling. It is commonly accepted that there are many uncertainties in these systems, with quantities such as ion channel kinetics or expression levels being difficult to measure or variable between samples. Until recently, the original approach of describing model parameters using single values has been retained, and consequently the majority of mathematical models in use today provide point predictions, with no associated uncertainty.

In recent years, statistical techniques have been developed and applied in many scientific areas to capture uncertainties in the quantities that determine model behaviour, and to provide a distribution of predictions which accounts for this uncertainty. In this paper we discuss this concept, which is termed uncertainty quantification, and consider how it might be applied to cardiac electrophysiology models.

We present two case studies in which probability distributions, instead of individual numbers, are inferred from data to describe quantities such as maximal current densities. Then we show how these probabilistic representations of model parameters enable probabilities to be placed on predicted behaviours. We demonstrate how changes in these probability distributions across data sets offer insight into which currents cause beat-to-beat variability in canine APs. We conclude with a discussion of the challenges that this approach entails, and how it provides opportunities to improve our understanding of electrophysiology.

## Highlights

• Uncertainty and variability in action potential models can be quantified.
• A probabilistic method for inferring maximal current densities is developed and applied.
• We use this to infer the currents responsible for canine beat-to-beat variability.
• Emulation of mathematical models provides rich information at low computational cost.
• The importance of considering uncertainty and variability in future is discussed.

## 1 Introduction

Models of the cardiac action potential (AP) are established, valuable, and important research tools because they integrate biophysical mechanisms quantitatively, and so have explanatory and predictive power. Since the publication of the first cardiac AP model over 50 years ago [31] these models have become more detailed as our knowledge of the function of ion channels, pumps, and exchangers in cardiac myocytes has increased [11]. Contemporary models are sufficiently detailed to allow the effects of ion channel gene mutations, pharmaceuticals, and disease to be examined in mechanistic detail [41], [28]. However, while the present generation of models are powerful tools, model parameters are generally assigned a fixed value, which means that the models produce a fixed prediction.

In contrast, the experimental APs recorded from real cardiac cells are variable, with changes from beat-to-beat in a single cell (termed intrinsic variability), and from one cell to another (extrinsic variability). Intrinsic variability may be caused by random processes such as stochastic ion channel gating, non-linear dynamics such as alternans of action potential duration (APD), or more complex behaviour. Extrinsic variability is considered to be caused by quantities that genuinely vary from cell to cell, e.g. cell size or ion channel expression. In practice it can be difficult to distinguish these sources of variability; in what follows we model variability as extrinsic only, although some of our data may also capture intrinsic variability. In addition, variability can be compounded by measurement errors when data from experiments are used to generate parameters for use in AP models.

Problems related to uncertainty and variability are not unique to cardiac electrophysiology, and new approaches are beginning to emerge from areas as diverse as models of the atmosphere [24] and galaxy formation [47]. In this paper we describe how these approaches might be applied to cardiac AP models.

### 1.1 Uncertainty quantification and cardiac action potential models

There are several potential sources of uncertainty in a computational model of a real system; these include, at least, the following [47]:

• *Observational uncertainty* is uncertainty or measurement errors in experimental data. For example, uncertainty represented by error bars in measurements of the current–voltage profile for a particular ion channel used to assign model parameters, or error bars in measurements of APD restitution used to evaluate model performance. Note that this uncertainty can encapsulate both intrinsic and extrinsic variability.
• *Parameter uncertainty* refers to uncertainty in model parameters, which may be a consequence of observational uncertainty as well as variability, or simply lack of information. It may be advantageous to express a model parameter (such as a maximal conductance) as a random variable with a distribution, rather than a fixed value.
• *Condition uncertainty* describes our uncertainty about the initial conditions and boundary conditions. For a cardiac AP model the initial conditions are typically set by running the model until it has reached a steady state, but this will not capture the constantly varying environment of temperature, ion concentrations, and metabolism in which a real cell operates.
• *Structural uncertainty* accounts for the differences between a model and the real system that it represents. For example, a model of an ion channel will not be an exact representation of the biophysical dynamics of a population of proteins in the membrane, and structural uncertainty aims to quantify this difference.
• *Simulator uncertainty* addresses the uncertainty introduced when using an approximation to the true solution of the equations of the mathematical model when we perform a simulation. This includes any uncertainty introduced by using discretisation in numerical methods (numerical error), or uncertainty when a fast-running surrogate model (e.g. an emulator) is used approximate the outputs of a computational model that is expensive to solve.

Techniques for uncertainty quantification (UQ) provide a means to deal with these different sources of uncertainty. In this paper we concentrate on UQ methods that address parameter and condition uncertainty, which will also concern observation and simulator uncertainty. Statistical methods for structural uncertainty can be complex and are outside the scope of this paper. Such techniques attempt to statistically quantify the ‘model bias’, the difference between model and experiment; the interested reader may refer to [22].

There are two stages to UQ related to parameter/condition uncertainty (for clarity we only refer to parameters below, but the same ideas apply to initial or boundary conditions, though these may be more difficult to measure):

1. *Uncertainty characterisation* regards uncertainty in model *inputs*. In this stage uncertainty in parameters is characterised by assigning probability distributions to input parameters instead of single values, although sometimes simple statistics (i.e. means and variances) are used. If the input is a parameter that is directly measurable, this is a purely experimental task because the probability distribution is informed by the experimental observations. On the other hand, if the input is a parameter that is indirectly inferred from other data, statistical methods may be required to estimate the parameter uncertainty (examples will be provided in this paper).
2. *Uncertainty propagation* (or *uncertainty analysis*) regards uncertainty in model *outputs*. Here the aim is to establish the uncertainty in model outputs due to the uncertainty in inputs, again as probability distributions or simple statistics. Generally, this stage is very computationally-demanding, since a large number of simulations are needed to generate outputs for the different combinations of inputs that are possible. Sophisticated methods have been developed to mitigate such difficulties, as will be illustrated in this paper

Interest in UQ has grown as part of a drive for rigorous and formal approaches to assess the credibility of computational models. The heavy use of computational models for safety-critical applications in the automotive, aerospace, nuclear and structural engineering industries in particular motivated the development of ‘Verification, Validation and Uncertainty Quantification’ (VVUQ), which forms a set of methodologies, frameworks and best practises for improved assessment of the reliability and robustness of model predictions [30], [33]. In this context, *verification* is defined as the process of confirming that a computational model (software) correctly implements an underlying mathematical model, and validation compares a model's predictions with reality. Although UQ forms part of the overall VVUQ process, each of the stages are intertwined, and in particular UQ improves the ability to perform validation, since understanding the uncertainty in model predictions facilitates comparison with experimental results.

Until recently, VVUQ has not been a priority for cardiac modelling, because this type of model has not been widely used in high-risk or safety-critical applications. However, the present generation of cardiac AP models are sufficiently detailed that there is the prospect that they could be used as both as part of clinical applications and also for drug safety assessment. Both of these applications are safety critical. For clinical applications the model output could be guidance for ablation in clinical procedures, and the inputs would include personalised measures of tissue conductivity and anatomy [46]. For safety testing in drug development, the output could be a measure of action potential prolongation, and the inputs would include a quantification of the reduction of different ion currents as a function of compound concentration [27]. In both types of application it will be important to express a measure of confidence in the model outputs, given uncertainties and errors in the inputs. As a result, there has been growing interest and application of (VV)UQ in cardiac modelling [14], [10], [36], [37], [38].

**Fig. 1:**
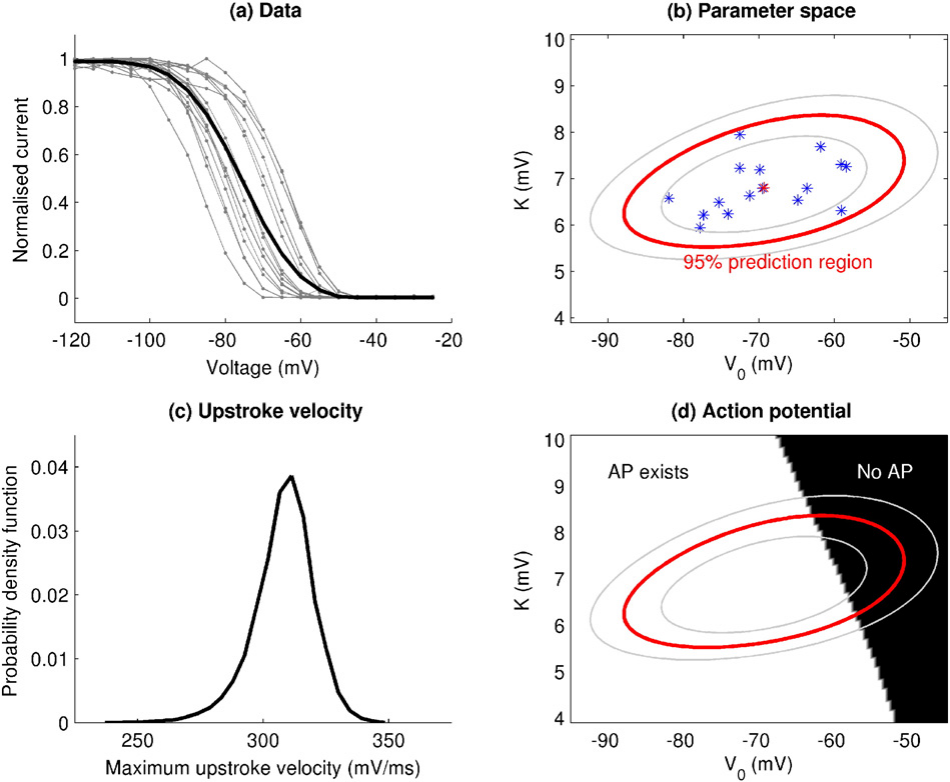
Pipeline of the UQ process, applied to variability in steady-state *I*_*Na*_ inactivation (image adapted from [38]). Using experimental data on steady-state *I*_*Na*_ inactivation from canine myocytes (sub-figure (a), individual cells in grey, averaged data in black, note how the slope of the average does not match the slope of any individual), uncertainty due to population variability was characterised by fitting a two-parameter sigmoidal curve using the statistical method nonlinear mixed effects to estimate the variability in the two parameters. Sub-figure (b) illustrates the mean (red star) and variability (red ellipse: 95%; grey ellipses: 80% and 99%) of the two parameters (blue stars represent parameters for individual cells). The mean corresponds to an ‘average’ cell, not the averaged data. This inactivation sub-model was then embedded in the Fox *et al.*[12] canine AP model, and the parameter uncertainty was propagated through the model to obtain a probability distribution for upstroke velocity (subfigure (c)). However, the model did not repolarise for a region of parameter space that overlapped with the population variability (subfigure (d)).

In [38] Pathmanathan *et al.* quantified the natural variability in the steady-state inactivation of the canine fast sodium channel using a statistical framework known as Non-Linear Mixed Effects (NLME) modelling. The authors examined the consequences of this uncertainty at the cellular and tissue scales, in perhaps the first application of uncertainty quantification to multi-scale cardiac modelling. In Fig. 1 we present a summary of this study, as it provides an excellent introduction to the concept of uncertainty quantification applied to this field.

We have also included a short extension to this work in Supplementary Material A, where we examine how the same technique can be applied to investigate both intra- and inter-animal variability in cellular recordings.

### 1.2 Aim and scope

The pipeline shown in Fig. 1 concentrates on a single component of *I*_*Na*_ channel behaviour, and examines how observation uncertainty can be expressed as parameter uncertainty, and how UQ can be used to establish how these uncertainties influence the model output.

The aim of this paper is apply a statistical UQ approach to cardiac AP models, and so gain mechanistic insight into the models as well as cardiac myocytes. We present two complementary case studies. In the first case study we show how the maximal conductances of ion channels can be inferred from noisy experimental recordings as distributions that express uncertainty about the estimates. The second case study then uses a statistical model (an *emulator*, surrogate model or metamodel) of a cardiac AP model to examine how uncertainties in maximal ion channel conductances influence uncertainty in model outputs, such as APD.

## 2 Case study 1: inference of maximal conductances from voltage recordings

Some of the most important parameters in determining AP model behaviour are the maximal densities of each ion current, which represent the number of each type of ion transporter in the cell membrane. In what follows we will use the terminology ‘conductances’ as shorthand to refer to the maximal densities of currents flowing through ion channels and also pumps and exchangers. In this case study we infer conductances for AP models from voltage vs. time data traces.

We expected that (i) the complexity of the model, and (ii) the richness of the experiment would determine the conductances that were constrained by data. For example, for a simple model with a small number (≤ 6) of ion currents, we might expect that only a certain combination of conductance values produces the observed AP, and therefore we can infer all of them. For a more complicated model with tens of conductances, there may be many options for ‘balancing the currents’ to give the same AP, and so we would not be able to establish unique estimates for each conductance. Similarly, a simple experiment such as a single AP recording may contain less information than a more complex protocol with, for instance, multiple pacing frequencies.

A similar approach was taken recently to estimate single ion channel kinetics [43], [42]. We consider a probabilistic method, and our overall aim is to compute the probability of any set of conductances (which we denote ***θ***) given the observed data, or *p*(***θ*** | data).

There are a number of experimentally plausible protocols that can be used to generate AP data, including steady pacing at different rates, changing ion concentrations of the extracellular solution, or applying a pharmaceutical intervention to block a specific ion current. We repeated the following study with a range of mathematical AP models, and a range of protocols, to examine how each affects the proportion of conductances that we can infer.

### 2.1 Methods

This case study was performed firstly on synthetic data, generated from a range of AP models, and then secondly on experimental canine action potential recordings.

#### 2.1.1 Synthetic data

We used noisy simulated data as our voltage recordings, which provide a best-case scenario of the recordings we would observe if the AP model was ‘correct’ (eliminating the need to consider structural uncertainty). We refer to these simulated traces with added noise as synthetic data. Synthetic data were generated by solving equations for a particular AP model, given one of our experimental protocols, with the conductances that feature in the published model. We sampled the simulation output at 0.2 ms intervals and then added random noise to each point of this synthetic voltage trace to simulate any noise in the system or the observations. The noise was randomly sampled from a Normal distribution with mean zero and standard deviation of *σ* = 0.25 mV, chosen as this described the noise at resting potential on a repolarised canine ventricular AP experimental recording (as described below). We then ‘forgot’ the conductances that were used ($\hat{\theta }$), and the noise parameter *σ*, and attempted to solve an inverse problem to infer these from just the synthetic data trace.

#### 2.1.2 Experimental data

Canine cardiomyocytes were isolated as described previously [29]. A drop of cell suspension was placed in a recording chamber, which was kept at 37 °C. After the cells adhered, perfusion with a normal modified Tyrode's solution was started. Cardiomyocytes were patched with patch pipettes with a resistance of 3–4 MΩ, and action potentials were recorded in whole-cell configuration. After obtaining access and adjusting series resistance compensation (> 70%), continuous 1 Hz pacing with a 3 nA, 2 ms stimulus was started. Time resolution of the recordings was 250 *μ*s (4 kHz), while voltage resolution was 31.2 *μ*V. Recordings in the dataset (available to download with the simulation code) cover ≈ 800 ms of each pace, when the action potential occurs, in order to give processing time to log these data continuously.

The bath solution was composed of (concentrations in mM) NaCl 130, KCl 4, CaCl_2_ 1.8, MgCl_2_ 1.2, NaHCO_3_ 18, HEPES 10 and glucose 10, and pH was set at 7.4 with NaOH. Pipettes were filled with (in mM) NaCl 10, KCl 130, MgCl_2_ 0.5, HEPES 10, MgATP 5, and pH was set at 7.2 with KOH. After 200 s of pacing 10 *μ*M of Moxifloxacin was introduced to the bath, and at 240 s the bath concentration of KCl was increased to 5.4 mM.

The APD of each experimental pace is shown in Supplementary Material Figure B2, along with an indication of the paces that were selected as approximately steady-state for training and validation purposes.

We chose to use the Davies *et al.* canine model [9] (a modified version of the Hund & Rudy model [20]) for fitting to these data, as its default parameters resulted in a 1 Hz action potential that displayed similar features to those observed in our experiments. To model the effect of adding 5.4 mM KCl we changed the extracellular K ^+^ in the model from 4 mM at control/training to 5.4 mM for predictions/validation. The action of 10 *μ*M Moxifloxacin was modelled as conductance block with IC_50_ values of: 29 *μ*M for I_Kr_ [1]; 206.7 *μ*M for I_Na_ [17]; and 158 *μ*M for I_Ks_ and I_CaL_ (both have pIC50 values of 3.8 in [27]). Further details on the methods, including treatment of the stimulus, can be found in Supplementary Material B5.

#### 2.1.3 Bayesian inference

Because all data are inherently noisy to some degree, it does not make sense to say that a model prediction ‘matches’ data, instead we wish to say ‘how close a match’ there is to data. Using a probabilistic framework we can assign a goodness-of-fit score called the likelihood to any set of conductances: the probability of the observed data arising if the conductances were the true values, i.e. *p*(data | ***θ***). Details of the mathematical definition of the likelihood and assumptions involved are provided in Supplementary Material B2; below we provide a brief summary.

To define the likelihood we need a statistical model for the noise associated with the recording. Here we used a simple framework given by:

(1) Observed data=System behaviour+Noise model.

Differences between simulated voltage trace and the observed (synthetic or experimental) data trace are then assumed to be due to noise, which we assumed to follow a Normal distribution with mean zero and an unknown standard deviation *σ*. (While *σ* = 0.25 mV was used to generate the synthetic data, in general this would not be known, and so we attempted to infer *σ* alongside the conductances.) The likelihood is computed by solving the model equations with some proposed parameter values and comparing the resulting voltage trace with the synthetic data trace. Assuming the measurements in the trace are independent, the total likelihood is the product of the likelihood of each point in the trace.^2^

Once the modelling phase has led to an expression for *p*(data | ***θ***), *inference* determines the probability of a parameter set given the data, *p*(***θ*** | data), also commonly denoted as *π*(***θ***). Bayes' Theorem yields.

(2) $$\pi \left(\theta \right)=\frac{p\left(\left(\text{data}|\theta \right)\right)p\left(\theta \right)}{\int p\left(\left(\text{data}|\theta \right)\right)p\left(\theta \right)d\theta },$$

where *p*(***θ***) is a probability distribution containing our prior knowledge and assumptions about the parameters, and the integral is over the whole range of possible parameter sets. We refer the interested reader to a good textbook introduction [39].

In this case, we supposed that we had very little prior information on the parameter values, and took a uniform prior over intervals ranging from 0.1 × to 10 × the original model value (i.e. *p*(***θ***) is equal to a positive constant within these intervals and zero otherwise). Note that current–voltage (IV) curves may provide more useful prior information for some ion currents where available.

With many parameters, we cannot directly compute *π*(***θ***), and so we use Markov Chain Monte Carlo (MCMC, [40]) methods, which only require being able to evaluate *p*(data | ***θ***) at any given parameter set ***θ***. MCMC methods output a sequence/chain of parameter sets that form approximate independent samples from our target probability distribution *π*(***θ***). For a more detailed description of the MCMC algorithm that we used, see Supplementary Material B.

As it approximates samples from *π*(***θ***), MCMC will spend time in regions of large likelihood, so we start MCMC from a point with a reasonably large likelihood. To this end, we first ran a minimisation algorithm on the negative likelihood. Here, we used a minimisation algorithm called Covariance Matrix Adaptation–Evolution Strategy (CMA–ES, [16]). CMA–ES was started from a random point within the prior intervals defining *p*(***θ***). This resulted in the point(s) with the best log-likelihood. We could then use one of these points as a starting point, and subsequently checked that MCMC visited the neighbourhood of all these points regularly, since approximately sampling from *π*(***θ***) requires sampling from all regions where *π*(***θ***) is large and smooth.

**Fig. 2:**
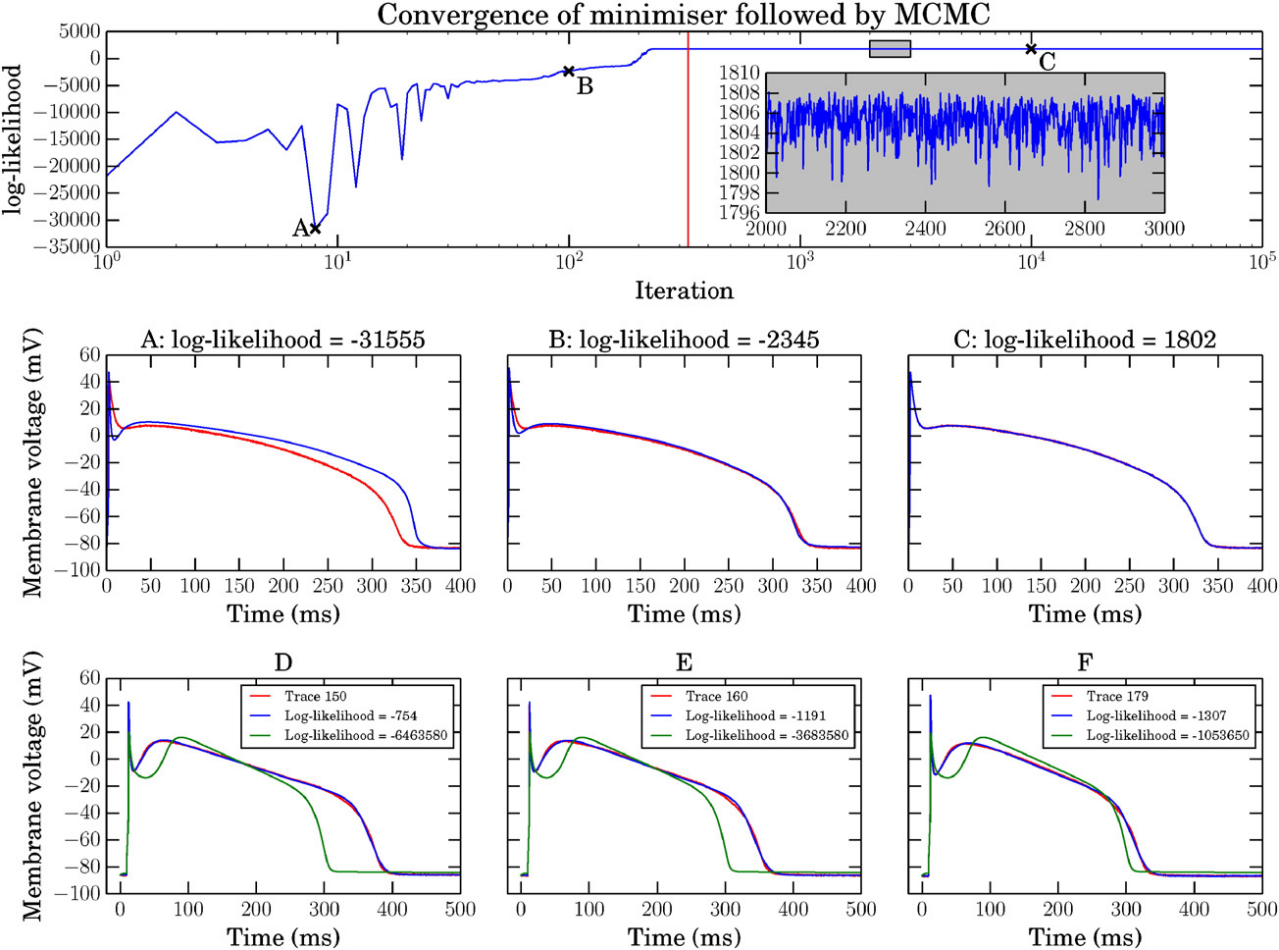
Top: synthetic data example of minimisation and MCMC for the Luo & Rudy model [25]: minimisation of the negative log-likelihood to find starting point for MCMC followed by log-likelihoods obtained during MCMC. The MCMC begins at the vertical red line, note the log scale on the *x*-axis — minimisation is a much smaller proportion of the iterations than MCMC. Points A, B and C are at iterations 8, 100 and 2000, respectively. Inset is a zoomed in view of the highlighted box, showing the MCMC exploring the likelihood between iterations 2000 and 3000. Panels A–C: the blue traces are the APs for the proposed parameters at iterations marked as A, B and C. Red traces are the same synthetic data in all 3 plots. Panels D–F: three experimentally recorded canine APs are shown in red in panels D, E and F. The green trace is the Davies et al. 1 Hz steady pacing AP [9], and the blue trace is the same model with conductances inferred to fit the experimental recordings.

Fig. 2 shows the likelihood of the parameter set during a CMA–ES run (mean likelihood given), followed by each MCMC iteration. Sets of parameters at three different points have been selected and their corresponding AP traces plotted over the experimental trace, to show the convergence of the minimisation and subsequent local search by MCMC.

After running the MCMC, we discarded the first quarter of all MCMC iterations as a *burn-in* period, during which we expect the chain to be settling into the target probability distribution. We also thinned the chain by only saving every tenth iteration. Practically, this reduces the output file size, and theoretically, it can help the chain better represent *independent* samples from the target distribution. The remaining samples form a distribution across parameters that approximates the probability distribution of conductances given the synthetic data.

All of the simulations were performed using the Chaste C++ library [26], [8]. The code we wrote for this is openly available, as described in Supplementary Material B1.

### 2.2 Results

#### 2.2.1 Synthetic Data

In this section we examine how much information on individual ion current conductances is present in synthetic data from a range of protocols, in terms of the certainty we gain in possible conductance values after performing the inference described above.

**Fig. 3:**
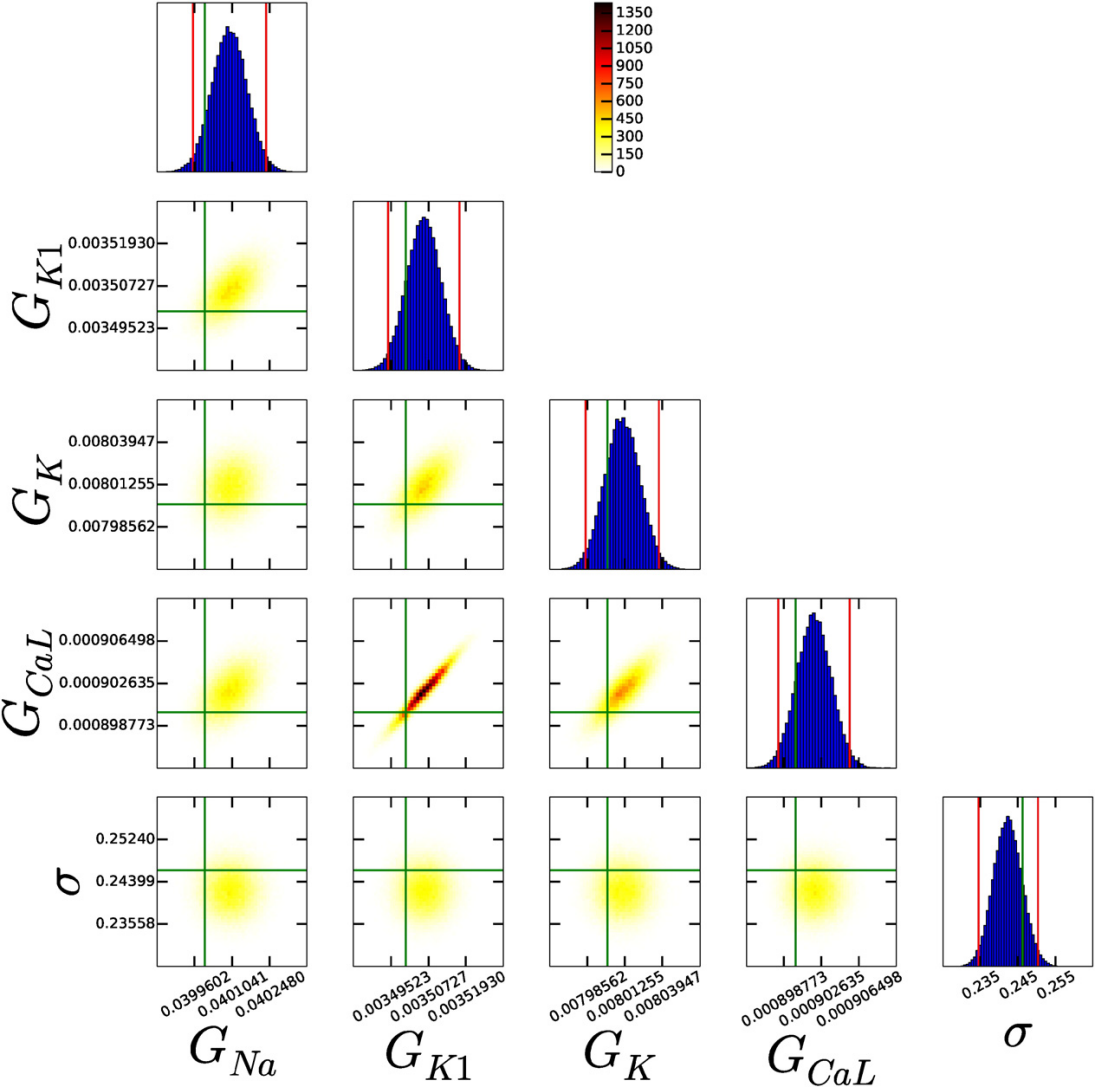
Marginal and 2-d normalised histograms representing marginal and pairwise probability distributions of each parameter, using the Beeler & Reuter model [3] model and single AP protocol. All parameters have been successfully inferred from the data (see main text). The vertical red (outside) lines give the 95% credible interval. The vertical green (inside) line is the parameter value used to generate the synthetic data.

The first experimental protocol we tested was 1 Hz pacing, recording the membrane voltage for sufficiently long to capture a single AP (as shown in Fig. 2A–C). As an example, Fig. 3 shows an array of scatter plots showing parameter distributions that were inferred for the Beeler & Reuter (1977) model [3]. In this plot the main diagonal contains histograms of the values each parameter takes through the MCMC run, and the off-diagonal entries contain heat maps showing the proportion of the run spent in that area for each pair of parameters. Also plotted are: the original parameter values, as vertical and horizontal green lines across the plots; and 95% credible intervals for each parameter as vertical red lines on the histograms (the intervals within which 95% of the values lie — constructed by ‘discarding’ the outermost 2.5% of the samples from the histogram, on each side).

In Fig. 3 all of the probability distributions are very narrow, in that the credible intervals (red lines) are very close, in absolute terms, to the original parameter values (green lines). We conclude that all 4 conductance parameters are successfully inferred from a single AP trace, and the noise parameter *σ* has also been inferred well.

To quantify this, we require a measure of how successful the inference process has been for any model and protocol.

For each parameter *θ*_*i*_, we define its mean absolute percentage error (*M*_*i*_) across the MCMC run (excluding the burn-in period) as:

(3) $$M_{i}=\frac{1}{N}\sum_{t=1}^{N}\left|\frac{{\hat{\theta }}_{i}-\theta_{t,i}}{{\hat{\theta }}_{i}}\right|,$$

where ${\hat{\theta }}_{i}$ is the actual value of the parameter, i.e. the value used when creating the synthetic data, *θ*_*t* , *i*_ is the value of parameter *i* at iteration *t* of the MCMC run, and *N* is the total number of iterations in the MCMC run. We say we have successfully inferred a parameter if *M*_*i*_ < 0.05 (on average the parameter value in the chain is within 5% of the actual value of the parameter).

A summary of the number of maximal conductance parameters successfully inferred for each model under the protocols is given in Table 1. The noise parameter *σ* is always successfully inferred, and therefore omitted from the table. A more detailed table of which conductance parameters were successfully inferred from the models ([44], [34] & [9]) is given in Table B1 in Supplementary Material B.

**Fig. 4:**
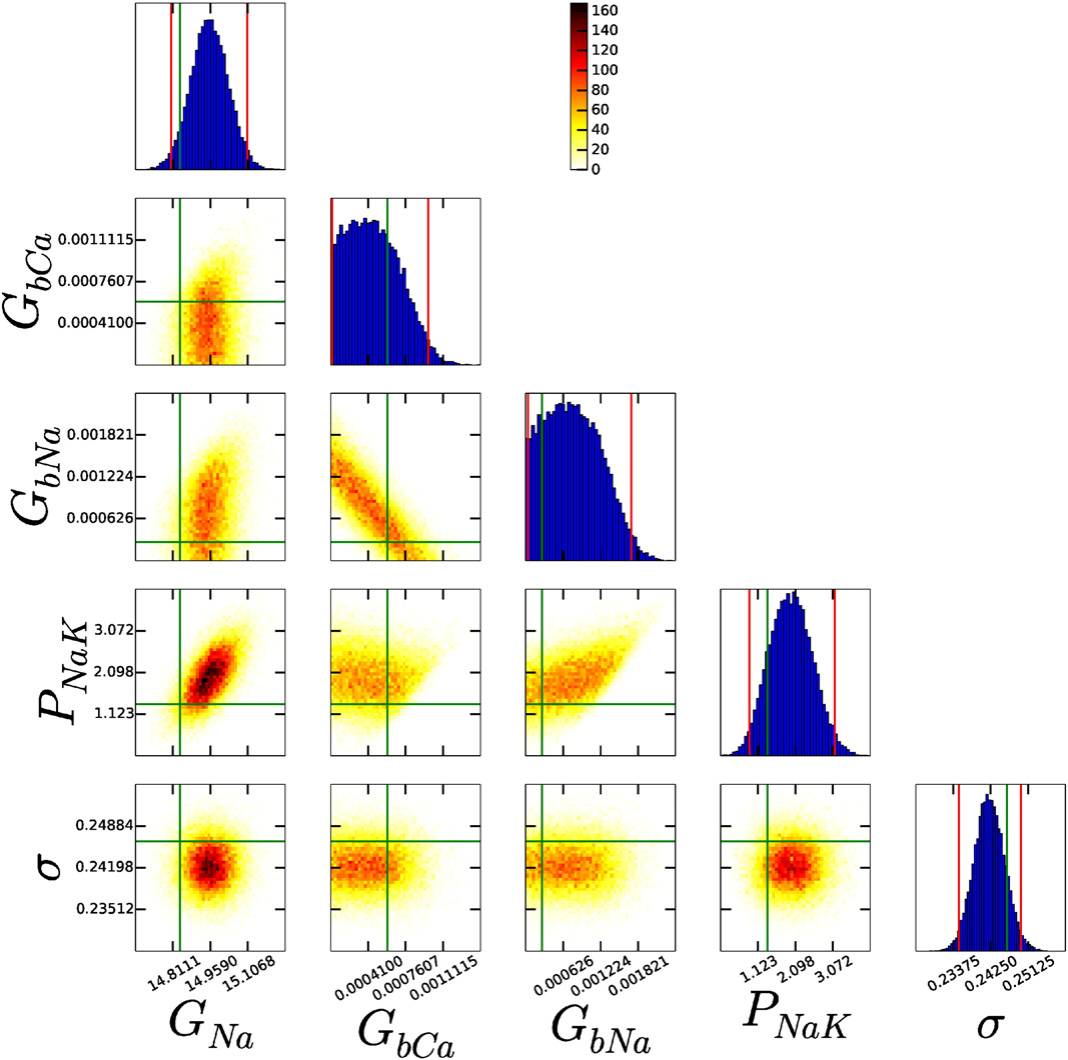
Subset of the histograms and pairwise probability distributions of each parameter using the ten Tusscher *et al.* (2004) model and single AP protocol for synthetic data. Not all parameters have been successfully inferred from the data, e.g. *G*_*bCa*_ , *G*_*bNa*_ and *P*_*NaK*_. *P*_*NaK*_ has the shape of a typically converged distribution, but fails our test of success by being too wide. The vertical red (outside) lines give the 95% credible interval. The green (inside) lines are the parameter values used to generate the synthetic data.

For the simple models [3], [19], [25] (with ≤ 6 conductance parameters), a single 1 Hz AP provides enough information for us to successfully infer all conductance parameters. However, a single AP did not provide enough information for us to successfully infer all parameters from the more complex models. For example, Fig. 4 shows a subset of the scatter plot array from the ten Tusscher *et al.* (2004) model [44]. Here, *G_Na_* is inferred successfully, and we see narrow histograms this parameter. However, the background currents *G_bCa_* and *G_bNa_* are not successfully inferred, and their histograms extend to the edge of the prior range we considered (0.1 × the original value). There is also a strong interdependence between the two parameters visible in their pairwise plot; it seems that a high value for *G_bCa_* and low value for *G_bNa_* fit the data just as well as a low value for *G_bCa_* and high value for *G_bNa_*. This dependence is an indication that there is unidentifiability of parameters — multiple parameter sets fit the data equally well, and the individual conductances cannot be identified (using this protocol). *P_NaK_* appears to have formed a well-defined distribution, but it is too wide to meet our criterion of success.

Other protocols shown in Table 1 include halving and doubling the extracellular potassium concentration, [K^+^]_o_, when measuring a single AP. The latter reduced the number of conductance parameters we could infer in the more complex models. In particular, using the ten Tusscher et al. [44] model and doubling [K^+^]_*o*_, the histograms for *G*_*K*1_, *G*_*Kr*_, and *G*_*Ks*_ are shifted to lower values (shown in Supplementary Material Figure B4). As such, the values moved away from the true parameters and so their *M*_*i*_ values increased. In an attempt to obtain a more informative voltage trace, we investigated longer and more complex protocols. We used an S1–S2 pacing protocol, 1 Hz followed by 2 Hz, and measured for a total of 2000 ms, giving a trace with three APs. In the case of the two most complex models, this increased the overall number of successfully inferred conductances, presumably as different currents give different contributions at the two rates, but we still could not infer all conductances.

We then tested a series of protocols in which we simulated the effects of pharmaceutical block of particular ion currents. With a 1 Hz pacing rate, we measured for a total of 2000 ms and therefore obtained 2 APs. At 1000 ms we set one maximal conductance parameter to zero, knocking out this current from the cell. Intuitively, one would expect such a protocol to give good information on the conductance of the current that was blocked, since it directly shows the effect on the AP of removing a particular ion current. The conductances we set to zero can always be inferred with these protocols, and in many cases the alteration in behaviour allows many additional conductances to be inferred as well (see Supplementary Material Table B1 for the full results).

The last protocols that were tested were 1 Hz pacing for 10,000 ms or 20,000 ms. These longer protocols allow slower time-scale dynamics to reveal their effects, and therefore for information about the conductances that govern these changes to be inferred more readily than from a single AP, for example the smaller background and pump currents. Somewhat remarkably, the inference process was able to successfully recover all 12 conductances in the ten Tusscher *et al.* [44] model, 11/13 in the O'Hara *et al.* [34] model, and 12/14 in Davies *et al.* [9] using just twenty 1 Hz APs. The two conductances in the O'Hara *et al.* [34] model which were not successfully inferred from the 20 s protocol are *G*_*bNa*_ and *G*_*pCa*_, and in the Davies *et al.* [9] model *G*_*bCl*_ and *G*_*pCa*_.

#### 2.2.2 Experimental data

As an example application of this technique we studied experimental canine action potentials gathered as described above in Section 2.1.2. It is well known that there is substantial variability in action potentials from beat-to-beat; but this behaviour is not very well understood and could be due to stochastic channel opening [13], turnover/regulation of ion channels, or chaotic dynamics [15].

**Fig. 5:**
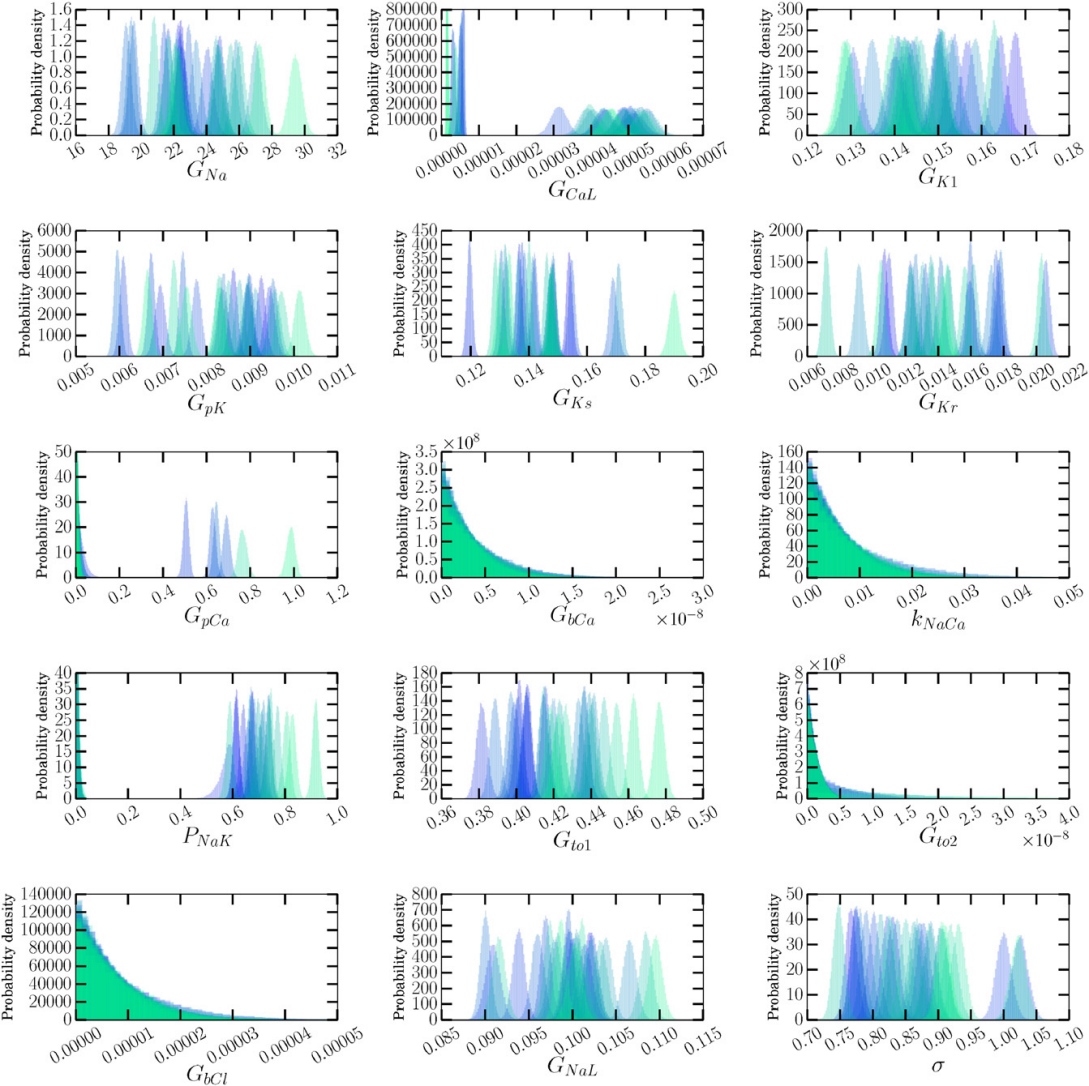
Posterior probability distribution estimates of current densities, and the noise parameter *σ*, inferred from a series of canine cardiomyocyte APs. While some currents are relatively consistent, e.g. *G*_*K*1_, others vary by a wide range, e.g. *G*_*Kr*_, *G*_*Ks*_. This figure should be considered in the light of the results in Table 1 (and Supplementary Material Table B1), which shows those currents for which we expect to be able to recover conductances using a single 1 Hz AP. The colours transition from semi-transparent blue  to semi-transparent green  through time.

We considered a recording from a single cardiomyocyte, and then inferred possible conductances for a single pace (in the control setting), allowing us to identify the majority of current densities for canine AP baseline to a high degree of accuracy (see Fig. 5). This process was repeated for a train of subsequent action potentials to uncover ionic currents that could be responsible for beat-to-beat variability within a single cell. While we do not believe that a sudden change of conductance values every second is a good model for the emergence of beat-to-beat variability in a single cell, it may be adequate to characterise the variability and generate a predictive model for it.

Fig. 2 panels (D–F) show three experimentally recorded APs (chosen to span the range of behaviours we observed), together with the [9] model AP with its original parameters for reference, and also with conductances adjusted to the maximum likelihood obtained of the inferred distributions. In all cases the inferred conductances result in a very good fit to the experimental AP compared with the default parameters, although some small differences are visible; for instance, the model fits all have a more pronounced change of gradient at the end of the action potential plateau than the experimental traces, which may indicate that the kinetics of the model's repolarisation currents need some refinement.

In Fig. 5 we present the conductances that were inferred for all membrane currents. Note that we do not expect all conductances to be fitted with narrow distributions to a single AP, as there was insufficient information in the synthetic data situation to recover them all perfectly for this model with its original conductances (see Table 1), so we do not expect to recover background, pump or exchanger conductances. These current densities are not directly accessible, and this is a novel way to infer the contribution of each ion current to the formation of the action potential and therefore normal cardiac function.

There are currents for which large differences are evident in the distributions inferred for each pace, that were all recovered with *M*_*i*_ < 0.25 in the synthetic data case. Observing distinct probability distributions between paces suggests that those currents take different magnitudes between different paces. For instance, *G*_*Ks*_, *G*_*Kr*_, *G*_*Na*_, *G*_*CaL*_ and *G*_*to*1_ all show large variation in Fig. 5, and are likely to play a large role in generating beat-to-beat variability. Interestingly, these same currents were identified as possible causes of beat-to-beat variability (also in a canine myocyte simulation study) when the currents were replaced with stochastic formulations ([18], see Fig. 2).

We also used the inferred conductances to predict the beat-to-beat variability that would be observed after Moxifloxacin addition and the change in bath concentrations; full results are shown in Supplementary Material B6. We predicted the correct trends in the data with an increased beat-to-beat variability in APD after the intervention, although the APDs themselves were generally shorter than those recorded experimentally.

## 3 Case study 2: GP emulators for cardiac action potential models

The analysis described above demonstrates how model inputs and parameters can be inferred as distributions, which embed uncertainty about the parameter value. In our second case study, we show how uncertainty in model parameters can be propagated through a cardiac AP model, so that we can see the effect of parameter uncertainty on the model output.

One approach to this question is to run the cardiac model many times, and to select parameter values for each run from the parameter distribution. Such Monte Carlo approaches require many model runs (typically several thousand) to build up distributions of the model output [7], and so in this case study we illustrate the use of statistical emulators (metamodels or surrogate models) for UQ in cardiac AP models, by building emulators of the ten Tusscher *et al.* (2006) model [45] (TP06) of the human ventricular myocyte.

An emulator is a statistical model that estimates the output of a computational model given a set of inputs. In our case the model outputs are descriptors of AP shape obtained from the TP06 model, and the inputs are the conductances of ion channels, pumps, and exchangers. An emulator approach therefore offers a way to explore the behaviour of a model without the cost of running a computationally expensive model many times. Emulators are trained using a set of design data, which are inputs and outputs obtained from runs of the computational model. The emulator can then be used as a fast-running surrogate to estimate model output for any set of inputs. The emulator also returns a variance function, which is a measure of the emulator uncertainty, and for example will be zero at the design points, where the true model output is known. For a graphical example of an emulator of a simple one-parameter model, see Supplementary Material C1.

A Gaussian Process (GP) emulator can use Normal distributions to represent both inputs and outputs, and so uncertainty is embedded throughout. An enormously powerful feature of GP emulators is that under certain assumptions, including that model inputs follow a multivariate Normal distribution, the corresponding distributions for model outputs can be computed analytically (see Supplementary Material C1 for an illustration). Therefore, uncertainty propagation, as well as variance based sensitivity analysis (see below) can be calculated directly from emulator properties—without repeated runs the of the emulator, let alone the original model.

### 3.1 Methods

Our approach to emulator construction was based on that described in previous studies [32], [24] and used a mathematical tool kit developed for UQ in computer models (http://mucm.aston.ac.uk/toolkit/index.php). For a detailed treatment of these techniques applied to a cardiac action potential model we refer the reader to an earlier study [7], and Supplementary Material C.

#### 3.1.1 Implementation of the TP06 model

The TP06 model for epicardial cells was implemented in MatLab (Mathworks), with code automatically generated from the Physiome Repository (http://models.physiomeproject.org, [48]). The model was configured to run for 20 beats at a cycle length of 1000 ms using the initial conditions specified in CellML, and was solved with the Matlab time adaptive solver for stiff systems of ordinary differential equations ode15s. Each beat was elicited with a stimulus of strength − 52 pA pF^− 1^ applied for 1 ms. This protocol enabled the model to approach a steady state, and in the multiple runs undertaken for the design data (see below) the greatest absolute difference in APD between the 19th and 20th beats was less than 0.5 ms.

#### 3.1.2 Inputs and outputs

We selected as model inputs the 12 maximal conductances in the TP06 model, along with a multiplier of the time constant for slow inactivation of the L-type Ca^2^ ^+^ channel *τ*_*f*_. The rationale for selecting these parameters as inputs was that the maximal conductances provide a clear link to cellular physiology as they represent the density of ion channel proteins in the cell membrane. As outputs, we selected six descriptors of AP shape based on membrane voltage (V_m_) measurements used in recent studies (e.g. [6]); maximum dV_m_/dt, maximum V_m_, dome V_m_, APD_90_, resting V_m_, and APD_50_. For the design and test data (see below) these outputs were obtained from the 20th AP in each run.

#### 3.1.3 Design data and emulator construction

We built separate emulators for each model output. The GP parameters for each emulator were determined by fitting to design data, comprising a set of input parameters and corresponding model outputs obtained from multiple runs of the model. For each model run, we selected the input parameters using Latin hypercube sampling from uniform distributions centred on the input parameter value specified in the original model, and with a range of half this value (see Table 2). We chose this range so that the emulator could be trained over a region of parameter space that was large enough to examine the effects of uncertainty in the input parameters. For clarity and convenience, each of these input parameter ranges were transformed into normalised units, so that the minimum value of the input parameter became 0, the maximum became 1, and the central value 0.5.

**Fig. 6:**
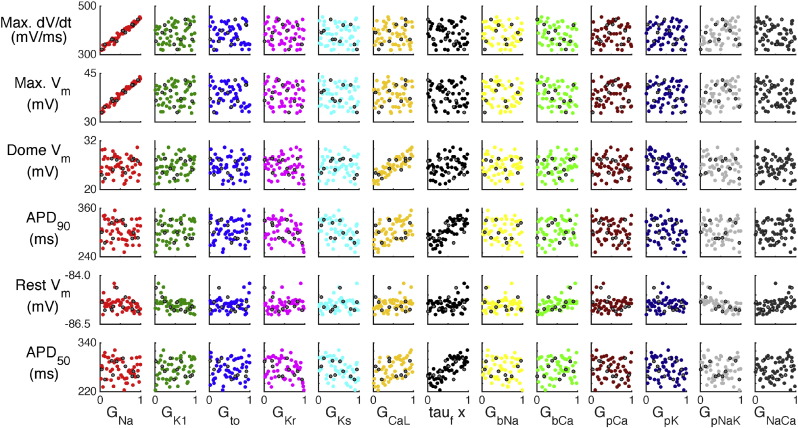
TP06 design data for 50 model runs (coloured circles), and test data obtained from 10 additional model runs (grey circles).

Design data from 50 model runs are plotted in Fig. 6, some dependencies are immediately clear. For example, *G_Na_* has a strong influence on both maximum dV_m_/dt and maximum V_m_, while *G_CaL_* has an influence on dome Vm. Both of these dependencies might be expected from knowledge of the physiology that the model represents. However many of the other associations between inputs and outputs are unclear from this figure, for example the model parameters that determine APD_90_ are not easy to identify.

The number of simulator runs in the design data needed to produce a good emulator can be determined by measuring the quality of the emulator fit with different numbers of design data. Quality of fit can be assessed using a further set of model runs (test points), where for each set of inputs the output of the emulator is compared with the output of the model using a statistical test [2]. We built emulators using design data from 25, 50, and 100 model runs, with an additional 10 model runs to produce test data. After comparing emulator outputs and test data outputs (see Supplementary Material C3 for the details), we established that 50 design points were enough model runs to obtain a satisfactory emulator fit for the TP06 model outputs, and these emulators were used for the results presented below.

### 3.2 Results

#### 3.2.1 Sensitivity analysis

Sensitivity analysis is a measure of how the output of a model depends on its inputs. The GP emulators built in this study can be used in two ways to assess how the model outputs depend on parameters.

First we assessed the contribution of each input to each output in mean effect plots, which show how the expectation of each output changed as each input in turn was assigned a fixed value that varied across the range 0 to 1 in normalised units (see Table 2 for this range in natural units), while the other inputs were assigned a fixed mean of 0.5 and a fixed variance of 0.04. We then calculated the main effect index, which for each input was the ratio of the variance in the mean effect when that input was fixed to 0.5 to the variance in the emulator output when all inputs were assigned a mean of 0.5 and a variance of 0.04. This quantity can be interpreted as a sensitivity index, which is the proportion of the total output variance that is accounted for by the variance on each of the inputs.

**Fig. 7:**
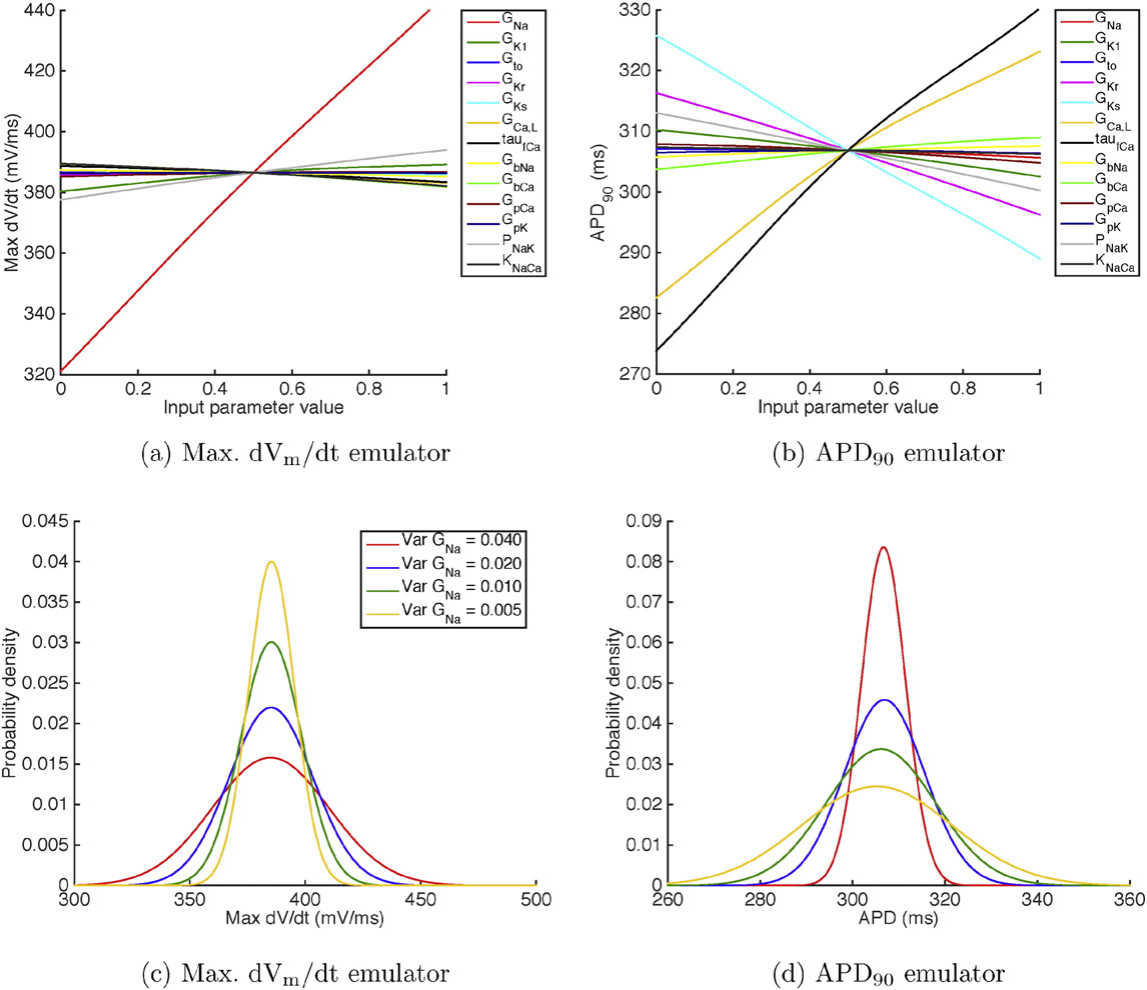
(a, b) Mean effects plots for (a) Max. dV_m_/dt and (b) APD_90_ emulators, showing how varying each input in the range from 0 to 1 influences the expected value of the emulator output. (c, d) Cumulative effect of changing the variance of inputs on emulator outputs. (c) Distributions of Max. dV_m_/dt when *G_Na_* was reduced from 0.04 (red), to 0.02 (blue), 0.01 (green), and 0.005 (yellow) while the variance of all other inputs was maintained at 0.04 normalised units. (d) Distributions of APD_90_ when the variance of all inputs was initially set to 0.001 normalised units, and then the variance of *G_Kr_* (red), *G_Ks_* (blue), *G_CaL_* (green), and then *τ*_*f*_ multiplier (yellow) were set to 0.04 normalised units.

The mean effects calculated directly from the Max. dV_m_/dt and APD_90_ emulators constructed from 50 design points are shown in Fig. 7(a) and (b) respectively. These plots show how the emulator output changed when each of the inputs in turn was varied in the range from 0 to 1 in normalised units (see Table 2 for these ranges in natural units), while the other inputs were effectively held constant at 0.5 by setting a variance of 0.04. When each parameter has a normalised value of 0.5, the emulator output was the same as the model output for the default parameter settings.

The overall pattern in these mean effects plots was consistent with the representation of cellular electrophysiology in the TP06 model and the design data shown in Fig. 6. *G_Na_* was strongly linked to Max. dV_m_/dt, whereas APD was influenced by the balance of K ^+^ and Ca^2^ ^+^ currents during the AP plateau and repolarisation. This observation confirms that the emulators have captured what we know about the model. However, these plots also quantify the effect of each parameter and so provide a quantitative mechanistic insight into the model as well as the physiology that the model represents.

**Fig. 8:**
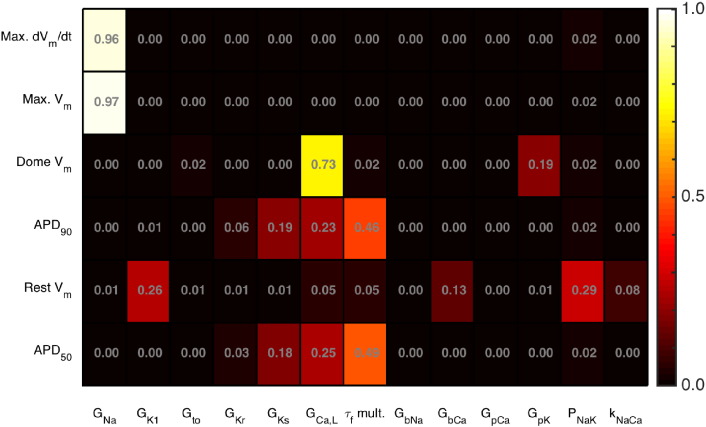
Main effect sensitivity indices for each TP06 emulator.

The main effects sensitivity analysis generalises the results from mean effects plots, and is shown in Fig. 8 for all of the inputs and each of the emulators. This measure indicates the proportion of the variance in each output (row) that can be attributed to variance in the input (column), and each square is coloured to indicate the magnitude of this index. As well as the links already shown in Fig. 7, these sensitivity indices show that Max. V_m_ is sensitive to *G_Na_*, *G_CaL_* influences Dome V_m_, APD_90_ and APD_50_ are both influenced by a similar group of inputs, and resting voltage is influenced by *G_K1_* and pump currents.

#### 3.2.2 Uncertainty propagation

To illustrate how a GP emulator can be used to examine uncertainty propagation, we calculated distributions of Max. dV_m_/dt and APD_90_ directly from the emulators, in cases where the model inputs were assigned different distributions. The details of these calculations are described elsewhere [32], [7].

Fig. 7(c) shows the effect of reducing the variance of *G_Na_* on the distribution of Max. dV_m_/dt. Initially, all of the inputs were assigned a mean of 0.5 and a variance of 0.04 in normalised units, so the mean of each input corresponded to the value in natural units given in the third column of Table 2. The distribution of Max. dV_m_/dt for these initial inputs is shown by the red line. The variance of *G_Na_* was then progressively reduced from 0.04, to 0.02, 0.01, and 0.005 in normalised units (0.60, 0.30, 0.15, and 0.075 nS pF^− 1^), and the resulting distributions of Max. dV_m_/dt are shown by the blue, green, and yellow lines respectively.

Reducing the variance of *G_Na_* can be interpreted as learning the value of *G_Na_* with greater confidence, and so this figure shows how uncertainty in the model output can be reduced by knowing the value of an input more precisely, using the approaches described in case study 1. However, the uncertainty in the output is only influenced by inputs to which the output is sensitive. Reducing the variance of *G_Na_* from 0.04 to 0.005 reduced the standard deviation of Max. dV_m_/dt from 25 to 10 mV ms^− 1^, whereas reducing the variance of *P_NaK_* from 0.04 to 0.001 had a minimal effect, reducing Max. dV_m_/dt by only 0.001 mV ms^− 1^.

In Fig. 7(d), we show the results of a multivariate analysis where we examine the effect of uncertainty in several inputs on uncertainty in APD_90_. Initially, all inputs were assigned a mean of 0.5, and a very small variance of 0.001. This configuration yielded an output distribution of APD_90_ with a mean of 306.7 ms and a standard deviation of 2.6 ms, which can be interpreted as a situation where the model inputs are known with confidence.

Experimental data [45] indicate variability in measurements of APD, and typically this variability is around 10 ms around the mean value. To illustrate how an emulator could be used to investigate the possible sources of variability in APD_90_, we increased the variance of some inputs from 0.001 to 0.04 and calculated the resulting distributions of APD_90_. Fig. 7(d) shows the effect of increasing the variance of *G_Kr_* (red line), *G_Kr_* and *G_Ks_* (blue line), *G_Kr_*, *G_Ks_*, and *G_Ca__L_* (green line), and *G_Kr_*, *G_Ks_*, *G_Ca__L_*, and *τ*_*f*_ (yellow line).

This illustrative example shows how an emulator can be used to identify the uncertainties in combinations of inputs that are consistent with uncertainty observed in outputs.

It should be stressed that this rich information has been gained from only 50 model runs for design data and 10 model runs for test data, many fewer than would be needed for Monte Carlo analysis. In a previous study [7], equivalent distributions of APD_90_ were calculated using an emulator and a Monte Carlo approach. Whereas multiple distributions could be calculated directly from the emulator trained on a single set of design data, 2000 model runs were required for *each* distribution calculated using a Monte Carlo approach.

## 4 Discussion

Models of the cardiac action potential are powerful tools that have provided mechanistic insight into complex biophysical systems. However, cardiac cells and tissue are adaptive and heterogeneous living systems, where there is variability in the shape and size of individual cells, and also in the density of ion channels, pumps, and exchangers in the cell membrane. Beat-to-beat fluctuations in ion concentrations and ion channel gating introduce further variability into the action potential. Although the present generation of cardiac action potential models can provide a mechanistic description of action potential generation, they neither capture these types of variability, nor is it always easy to determine how the model outputs depend on the parameters.

In the case studies presented here, we have shown how mathematical, statistical and computational techniques for uncertainty quantification can be adapted for use in cardiac electrophysiology modelling. These powerful techniques can be used to quantify uncertainty when fitting parameters to data; and then to examine the consequences of this uncertainty on model predictions, either by running a simulation many times or by building a simpler statistical model (emulator) of a biophysical model. UQ allows us to place confidence on model predictions, and hence is a first step in allowing us to say how much trust we should place in model predictions. A better understanding of uncertainty in cardiac action potential models will yield new insights into the sources of variability, its mechanism(s) and consequences, in real cardiac cells and tissue.

To explore UQ applied to cardiac AP models, we have presented two case studies which illustrate some of the key aspects. We can gain an understanding of the challenges faced by considering future extensions of these studies.

Case Study 1 illustrated how conductances can be inferred, with uncertainty, from AP recordings using a Markov Chain Monte Carlo (MCMC) inference method. This method was initially tested using synthetic data, and used to investigate the experimental protocols necessary to identify conductances using a variety of cardiac AP models. Importantly this study also demonstrated how a protocol may not provide sufficient information to constrain certain parameters; uncertainty propagation should be used to ascertain whether this is a problem for making predictions in any given context. The method has important potential applications in model development and optimal experimental design.

We applied the method to real experimental AP recordings, where we also examined possible causes of beat-to-beat variability. Parameter distributions were inferred from each individual voltage trace for a given pace. Plotting the distributions for a single parameter from multiple different AP traces together shows the beat-to-beat variability in maximal ion channel conductances. This is a new method for investigating which currents underlie beat-to-beat variability, providing novel insight into normal cardiac function. In particular, *G*_*Kr*_ has amongst the widest spread of distinct probability distributions, suggesting that *I*_*Kr*_ is one of the main currents responsible for beat-to-beat variability. The variation in maximal ion current conductance represents the varying number of active ion channels in the membrane throughout each pace. The spread of distributions for *G*_*Kr*_ suggests that this number of active channels could vary by up to a factor of 3. Similarly, the spread of distributions for *G*_*Na*_ suggests that the number of active sodium channels may vary by a factor of 1.5. Whether these fluctuations are due to stochastic opening of ion channels, or other regulation/kinetics operating over multiple paces, will require further investigation.

Case Study 2 demonstrates the power of Gaussian Process (GP) emulators. Emulators (fast-running surrogates of computationally-demanding models) in general, will be important for uncertainty propagation and sensitivity analysis, since they enable exploration of how outputs depend on multiple possible parameter values. GP emulators are especially powerful because under the assumption that input parameters can be described by a (multivariate) Normal distribution, output distributions for uncertainty propagation and sensitivity analysis can be computed directly. However, an important assumption of GP emulators is that the output surface is smooth. The good fit of the emulators in this study, evidenced by the Mahanalobis distance (Supplementary Material C3) indicates that for the TP06 model under steady pacing this is a reasonable assumption. Emulators of models with more complex behaviours such as APD alternans at short cycle lengths, will therefore need to be constructed carefully. In this case study input parameter uncertainty was not estimated from data. In future work, full conductance-related UQ could be performed by using the results of Case Study 1 as input distributions for the Case Study 2 emulator, although if these input distributions are not well-approximated as multivariate Normal distributions then repeated sampling using the emulator would be required.

In Pathmanathan *et al.* [38], uncertainty due to natural variability in steady-state *I*_*Na*_ inactivation was characterised, and the resultant effect on outputs of an AP model containing the inactivation sub-model computed. The characterisation of this variability could be improved by developing a better understanding of inter- vs. intra-animal variability (as discussed in Supplementary Material A), and other sources of variability. Repeating this process for other sub-models of cardiac AP models will require significant experimental and modelling effort.

### 4.1 Other sources of uncertainty

In this article we have focused on uncertainties in ion channel and action potential model parameters. When moving to larger spatial scales, using mono/bi-domain partial differential equation models for spatial as well as temporal movement of charge, a number of other sources of uncertainty will be introduced. One of the major influences at this scale is the spatial distribution of action potential and ion current properties. For example, simulation study findings could be strongly influenced by trans-mural, apex-base, and left–right changes in ion channel expression in the ventricular walls, as well as variations in these properties throughout the septum, papillary muscles, atria and conduction system.

Some important work has begun on examining the influence of spatial uncertainties, for example how different possible underlying patterns of *I_Ks_* expression influence T-wave morphology [21]. In another recent example, a whole-heart simulation study found arrhythmias were inducible under simulated application of 8 drugs associated with clinical risk, but when changing the spatial distribution of action potential model properties, to remove a distinct population of ‘M cells’, arrhythmias were no longer inducible for any compounds [35].

Other tissue-scale parameters that are associated with substantial uncertainty include the degree and orientation of cell-to-cell coupling throughout the heart. The impact of other cell types, blood vessel distribution and fine-scale geometry are usually ‘homogenised away’ in modelling assumptions, but may also strongly influence findings [23], [4], [5]. As tissue mechanics, blood flow, and fluid/solid interaction are introduced into sophisticated electro-mechanical models, the uncertainties that are involved will rapidly escalate.

Much remains to be done to establish the sensitivity of any conclusions from tissue simulations to these uncertainties. The computational challenge is substantial, and will require technologies such as the emulator we introduced in this article to be applied to these problems. We expect that uncertainty analyses will be particularly important in terms of susceptibility to arrhythmias, which may inform clinical decisions in the future.

### 4.1 Conclusions

Uncertainties in cardiac action potential models and their parameters need to be carefully quantified, and that results from simulation studies should be examined in a probabilistic framework to establish the robustness of their predictions to uncertainties. Such uncertainty quantification is crucial to the development of robust, reliable and trustworthy electrophysiological models, since cardiac myocytes are associated with much observational uncertainty and natural variability, and UQ allows these to be formally and rigorously integrated into the models. While the statistical techniques applied in this paper are advanced, we wish to emphasise that in some cases the simplest approach to UQ may be sufficient (fitting a distribution for a parameter to a dataset, and then using repeated sampling to estimate the distribution of a model output, see [10] for an example of this). In general, however, a close collaboration between experimentalists, modellers and statisticians (amongst others) will be required.

Rigorous uncertainty assessment will be crucial for safety in clinical applications, but there are likely to be important benefits for basic science too. Formal UQ will improve our quantitative understanding of the sources and characteristics of natural variability in physiological systems, together with quantitative understanding of the consequences of such variability. Overall, UQ applied to cardiac action potential models will be tremendously important for developing trustworthy models *and* will provide a means for developing deeper physiological understanding of variability.

## Sources of Funding

RHJ is supported by a Systems Approaches to Biomedical Science Industrial Doctorate Centre studentship (EP/G037280/1) and F. Hoffmann-La Roche AG. RHC and ETYC gratefully acknowledge support from the UK Engineering and Physical Sciences Research Council (www.epsrc.ac.uk) grant number EP/K037145/1. GRM gratefully acknowledges support from a Sir Henry Dale Fellowship jointly funded by the Wellcome Trust and the Royal Society (Grant Number 101222/Z/13/Z).

## Conflicts of Interest

The authors declare that the research was conducted in the absence of any commercial or financial relationships that could be construed as a potential conflict of interest.

## Disclosure

The mention of commercial products, their sources, or their use in connection with material reported herein is not to be construed as either an actual or implied endorsement of such products by the U.S. Department of Health and Human Services.

## Figures and Tables

**Table 1:**
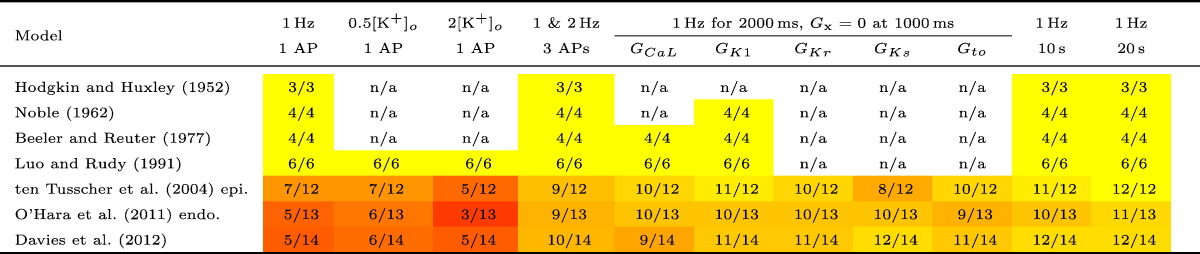
Number of conductance parameters that were constrained sufficiently given a particular model and simulated protocol. Our definition of success is that *M* < 0.05 for a parameter for the length of the MCMC chain (having removed the first quarter as a burn-in period). ‘n/a’ stands for ‘not applicable’, and indicates that the models do not include the components necessary to perform these protocols. N.B. *G*_*to*1_ appears in [9], but *G*_*to*_ is in all other models in which the current is modelled.

**Table 2:** Range of conductances used to fit the TP06 emulator with 25, 50 and 100 design points.

Input parameter	Units	Mean	Range
*G*_*Na*_	nS pF ^-1^	14.838	7.42–22.26
*G*_*K*1_	nS pF ^-1^	5.405	2.70–8.10
*G*_*to*_	nS pF ^-1^	0.294	0.147–0.441
*G*_*Kr*_	nS pF ^-1^	0.153	0.077–0.230
*G*_*Ks*_	nS pF ^-1^	0.392	0.196–0.588
*G*_*CaL*_	cm ^3^ ms ^-1^ *μ*F^-1^	0.0000398	0.0000199–0.0000597
*τ*_*f*_ multiplier	dimensionless	1.0	0.50–1.50
*G*_*bNa*_	nS pF ^-1^	0.00029	0.000145–0.000435
*G*_*bCa*_	nS pF ^-1^	0.00059	0.000295–0.000885
*G*_*pCa*_	nS pF ^-1^	0.123	0.0615–0.1845
*G*_*pK*_	nS pF ^-1^	0.0146	0.0073–0.0219
*P*_*NaK*_	pA pF ^-1^	2.724	1.362–4.086
*k*_*NaCa*_	pA pF ^-1^	1000.0	500.0–1500.0

## Appendix A Supplementary data

Additional methods and results can be found in this document.

## Footnotes

2 Since likelihoods are often the product of very small numbers, we generally compute the logarithm of the likelihood, which is less prone to numerical error, and for a Normal distribution simplifies the calculation. The log-likelihood can be positive or negative; a larger log-likelihood in the positive direction indicates a better fit.
