# Supplementary material for: Uncertainty and variability in models of the cardiac action potential: Can we build trustworthy models?
Source: J Mol Cell Cardiol. 2016 Jul;96:49–62. doi: 10.1016/j.yjmcc.2015.11.018 (PMC4915860; doi:10.1016/j.yjmcc.2015.11.018)
Supplement: Supplementary file 1 — Additional methods and results can be found in this document. [file mmc1.pdf]

Supplementary Material for: Uncertainty and variability in models of the cardiac action potential: can we build trustworthy models?

Ross H. Johnstone, Eugene T. Y. Chang, Rémi Bardenet, Teun P. de Boer,
David J. Gavaghan,Richard H. Clayton, Gary R. Mirams & Pras Pathmanathan

October 13, 2015

Contents

A Steady state I_{Na} inactivation: intra- vs. inter-animal variability **2**

B Case Study 1: Further Details **4**
B1 Numerical Methods 4
B2 Likelihood 4
B3 Pre-MCMC CMA–ES minimisation 5
B4 Adaptive Metropolis-Hastings algorithm 6
B5 Additional methods for simulation of canine patch-clamp experiment 6
B6 Additional results for Section 2.2 9
B6.1 Prediction of beat-to-beat variability in a canine myocyte 12

C Case Study 2: introduction to GP emulators **13**
C1 Simple example 13
C2 Emulator implementation 14
C3 Emulator validation 15
C4 Emulator Implementation 16

Supplementary Material

A Steady state $I_{\mathbf{Na}}$ inactivation: intra- vs. inter-animal variability

The results of Pathmanathan et al. (2015), as illustrated in Figure 1, use data from 16 canine endocardial myocytes, originally published in Cordeiro et al. (2008). These data were obtained from multiple animals in experiments that took place over the course of over one year. Seven animals contributed a single cell to the dataset, three animals contributed two cells, and one animal three cells. In Pathmanathan et al. (2015), inter-subject vs intra-subject variability was not assessed. Here we present a short preliminary analysis of inter- vs inter-subject variability, and discuss how this variability could be statistically quantified.

Figure A1, top-left, plots the same data as Figure 1, top-right, but with lines connecting cells from the same animal, so that inter- and intra-animal variability can be easily visualised. The remaining panels show the same results using three other canine datasets: data from 15 epicardial cells originally published in Cordeiro et al. (2008), and data from 10 epi and 10 endocardial cells originally published in Murphy et al. (2011). All four datasets were used in Pathmanathan et al. (2015). Note that the axis scales are quite different. For a discussion on the differences between epi- vs endocardial cells, and on differences between the Cordeiro et al. (2008) and Murphy et al. (2011) datasets, see Pathmanathan et al. (2015).

The results do not display obvious clustering for cells from the same animal, although a visual inspection suggestions correlation in V_0 and K for cells from the same animal cannot be ruled out. It is important to realise that the non-linear mixed effects (NLME) method that was used to analyse the data also provides a framework for including these different sources of variability. The function that was fit to the data was the sigmoid $F(V; V_0, K) = 1/(1 + \exp((V - V_0)/K))^2$. Let $\mathbf{q} = (V_0, \ln(K))$. The statistical model that was used in Pathmanathan et al. (2015) was

$$\mathbf{q}_i = \mathbf{q}^* + \mathbf{b}_i, \tag{A.1}$$

where i is cell index, $\mathbf{q}^*$ is the fixed effect (red star in Figure 1), and $\mathbf{b}_i$ is the random effect, corresponding to cell-to-cell variability. The $\mathbf{b}_i$ were assumed to be Normally distributed with mean zero and covariance matrix Ψ. The NLME procedure simultaneously estimates $\mathbf{q}^*$ and Ψ. This can be extended to account for inter- and intra-animal variability, for example by replacing the statistical model (A.1) with

$$\mathbf{q}_{ij} = \mathbf{q}^* + \mathbf{a}_i + \mathbf{b}_{ij}, \tag{A.2}$$

where i is animal index and j cell index, and where we could assume $\mathbf{a}_i \sim N(0, \Psi_a)$ and $\mathbf{b}_{ij} \sim N(0, \Psi_b)$. The NLME method can be used to estimate $\mathbf{q}^*$, Ψ_a and Ψ_b. More general statistical models are also possible, such as $\mathbf{b}_{ij} \sim N(0, \Psi_b(\mathbf{a}_i))$, which corresponds to the amount of intra-subject variability being subject-dependent.

Analyses such as these should provide deeper understanding of natural variability, although estimating the unknown quantities in the above formulation may require more data than presented in Figure A1, in which data from only a single cell is available for many of the animals. Other factors such as age and gender may also influence inactivation, and in addition Figure A1 shows that transmural location affects inactivation properties (see Pathmanathan et al. (2015)). These observations emphasise how developing a full quantitative understanding of natural variability, even just for this aspect of cardiac electrophysiology, is a major experimental and statistical task.

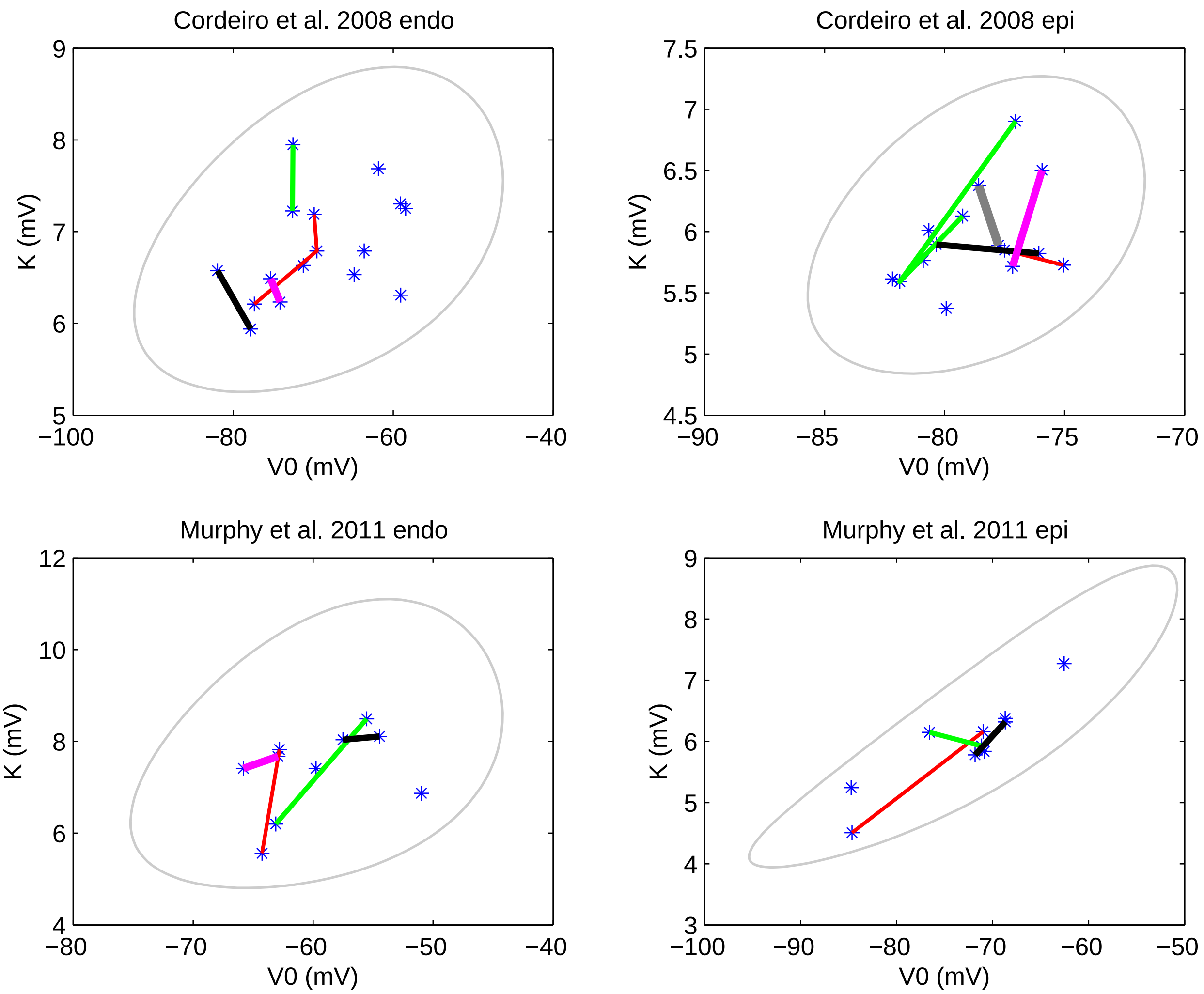

Figure A1: Results of the NLME fitting process for four separate canine steady steady I_{Na} inactivation datasets. Stars are the values corresponding to individual cells, the grey line is the 99% prediction region. Coloured lines connect cells which originated from the same animal. The colours are for ease of visualisation only, so for example the green line in the top-left sub-figure does *not* correspond to the same animal as the green line in the top-right sub-figure. Note the different axis scales.

B Case Study 1: Further Details

B1 Numerical Methods

The action potential models were downloaded in XML format from the CellML repository (Lloyd et al., 2008). The equations were converted into and solved in C++ using a CVODE solver within the Chaste framework (Mirams et al., 2013; Cooper et al., 2014). The relative and absolute tolerances for the CVODE solver were 10^{-7} and 10^{-9}, respectively. The sampling time of the CVODE solver was set to 0.2 ms. The maximum number of steps the CVODE solver can take in its attempt to reach the next output time was set to 5000. This last condition was set to solve a numerical problem we were having where the proposal traces had a 'notch' in them which made acceptance of new states very unlikely, and so the chain became stuck at some set of parameter values. Increasing the maximum number of steps smoothed out the trace for the same parameter values so that MCMC could proceed as normal.

The CMA–ES minimisation (Hansen, 2006) was implemented in Python. The objective function to be minimised was the negative of the log-likelihood returned by Chaste. The default options were used. The source code is available at `https://www.lri.fr/~hansen/cmaes_inmatlab.html`.

A custom written Python script was used to perform the MCMC. It interfaced with C++ by sending a set of parameters to Chaste and waiting for a log-likelihood to be returned by Chaste before performing the adaptive MCMC steps and moving onto the next iteration. The Python script uses the NumPy library (Van Der Walt et al., 2011). The figures were produced using `matplotlib` (Hunter, 2007).

A bolt-on project called `Jmcc_UQ`, compatible with the computational biology C++ library Chaste (v3.3), is available to download from `http://www.cs.ox.ac.uk/chaste/download.html`, it contains all the necessary code to reproduce Case Study 1.

B2 Likelihood

Given our simulated data, we compute the likelihood of observing this data given a certain set of parameters. We make the assumptions:

- The only source of error in our measurements is that of experimental noise. In other words, that the measured data is Normally distributed around some true value, with a standard deviation of σ mV.
- We treat σ as an unknown parameter to be inferred, along with the maximal conductance parameters.
- The values of the noise around the true solution at each time point are independently distributed.
- We have a rough idea of what the parameters are; prior knowledge can be accurately represented by a uniform distribution across large intervals (which we choose to be from 0.1 × (the true parameter value) to 10 × (the true parameter value)). Their joint prior distribution is given by Equation (B.3).

$$p(\boldsymbol{\theta}, \sigma) = \begin{cases} c & \{\boldsymbol{\theta}, \sigma\} \text{ in some hypercuboid,} \\ 0 & \text{otherwise,} \end{cases} \tag{B.3}$$

where c is a non-zero finite normalising constant.

We generate a test trace by solving the model equations with the conductance parameters $\boldsymbol{\theta}$. We then compute the likelihood of this test trace given our experimental trace using Equation (B.4),

which is the product of (independent) Normal probability density functions. $\boldsymbol{x}$ is the experimental trace vector and $\boldsymbol{f}(\boldsymbol{\theta})$ is the test trace vector. Both vectors have N entries.

$$L(\boldsymbol{\theta}, \sigma) = p(\text{data}|\{\boldsymbol{\theta}, \sigma\}) = \prod_{i=1}^{N} \mathcal{N}\left(x_i | f_i(\boldsymbol{\theta}), \sigma^2\right) = \prod_{i=1}^{N} \frac{1}{\sqrt{2\pi\sigma^2}} \exp\left(-\frac{(x_i - f_i(\boldsymbol{\theta}))^2}{2\sigma^2}\right). \tag{B.4}$$

We often deal with very small values close to zero, so we take the natural log of the likelihood, giving:

$$\begin{aligned} l(\boldsymbol{\theta}, \sigma) &= \log(L(\{\boldsymbol{\theta}, \sigma\})) \\ &= -\frac{N}{2}\log(2\pi\sigma^2) - \frac{1}{2\sigma^2}\sum_{i=1}^{N}(x_i - f_i(\boldsymbol{\theta}))^2 \\ &= -\frac{N}{2}\log(2\pi) - N\log(\sigma) - \frac{1}{2\sigma^2}\sum_{i=1}^{N}(x_i - f_i(\boldsymbol{\theta}))^2 \end{aligned} \tag{B.5}$$

Since the first term on the RHS of Equation (B.5) is constant for all proposed $\boldsymbol{\theta}$, it will cancel with itself when we take differences of log-likelihoods later on. It is therefore enough to define

$$l(\boldsymbol{\theta}, \sigma) \propto -N\log(\sigma) - \frac{1}{2\sigma^2}\sum_{i=1}^{N}(x_i - f_i(\boldsymbol{\theta}))^2. \tag{B.6}$$

where $\propto$ means "up to an additional constant". For notational simplicity, we concatenate $\boldsymbol{\theta}$ and σ into a single vector, which we will still call $\boldsymbol{\theta}$. We calculate the posterior probability density, $\pi(\boldsymbol{\theta})$, of a set of parameters $\boldsymbol{\theta}$ using Bayes' rule:

$$\pi(\boldsymbol{\theta}) = p(\boldsymbol{\theta}|\text{data}) = \frac{p(\text{data}|\boldsymbol{\theta})p(\boldsymbol{\theta})}{\int_{\boldsymbol{\Theta}} p(\text{data}|\boldsymbol{\theta})p(\boldsymbol{\theta})\, d\boldsymbol{\theta}}. \tag{B.7}$$

We are unable to integrate functions with respect to $p(\boldsymbol{\theta}|\text{data})$ given in Equation (B.7), so we sample from this distribution using MCMC to allow us to approximate these integrals.

B3 Pre-MCMC CMA–ES minimisation

Before starting our MCMC to sample from the probability distribution, we want some idea of where $\pi(\boldsymbol{\theta})$ puts mass in the parameter space Θ. We therefore run a CMA–ES (Hansen, 2006) minimiser with objective function $-l(\boldsymbol{\theta})$. This will hopefully give us a point estimate for the greatest log-likelihood. MCMC with a Gaussian proposal will work best if the mass is distributed around this point, and that there are no other modes elsewhere. If several runs of CMA–ES only find one point with the best log-likelihood, it is likely that the target distribution will be uni-modal.

We run the CMA–ES minimiser from a number of starting points in the unit hypercube. These starting points are randomly sampled uniformly from this space. These values are then linearly scaled to lie in the support of the prior given in Equation (B.3). The final values returned by the minimiser are saved, and the one yielding the best log-likelihood is used as the starting point for the MCMC, after being re-scaled. We have used the default options in CMA–ES, with an initial step size of 0.1.

B4 Adaptive Metropolis-Hastings algorithm

We sample from this distribution using MCMC with Metropolis-Hastings[1] with adaptive covariance matrix, given in Algorithm 1. For the first 1,000 × (number of parameters) iterations, there is no adaptation. This is to establish those directions in parameter space which are associated with good likelihood. After this, the covariance matrix of the multivariate Normal proposal distribution is updated at each iteration, taking into account the direction of accepted points. This adaptive covariance matrix aligns itself along directions with the best log-likelihoods, and a scalar multiple scales how wide the proposal distribution is.

After running the MCMC, we discard the first quarter of all the iterations as *burn-in*, during which time the chain is settling into its stationary distribution, which is our target distribution by construction. We let the MCMC run for long enough so that the initial stage of non-adaptivity is entirely contained within the burn-in and so is discarded.

We also perform *thinning* on the chain by only saving every 10th iteration. Practically, this reduces the output file size, and theoretically, this reduces auto-correlation of the samples, so that the chain intuitively better represents independent samples from the target probability distribution.

Algorithm 1 Adaptive Metropolis-Hastings MCMC, continued in Algorithm 2.

$\log(a_0) \leftarrow 0$.
$\boldsymbol{\theta_0}$ determined by CMA–ES minimisation.
$\mu_0 \leftarrow \boldsymbol{\theta_0}$.
$\Sigma_0 \leftarrow D$, where D is a diagonal matrix.
$t := 0$
while $t \leq 1000\times$(number of parameters) **do**
  Given the current parameter state $\boldsymbol{\theta_t}$, sample $\boldsymbol{\theta^*} \sim \mathcal{N}(\cdot|\boldsymbol{\theta_t}, a_0\Sigma_0)$.
  if $p(\boldsymbol{\theta^*}) \neq 0$ **then**
    Compute $\log(\alpha) = l(\boldsymbol{\theta^*}) - l(\boldsymbol{\theta_t})$.
    Sample $u \sim \mathcal{U}(0,1)$.
    if $u < \alpha$ **then**
      $\boldsymbol{\theta_{t+1}} \leftarrow \boldsymbol{\theta^*}$.
      accepted $\leftarrow 1$.
    else
      $\boldsymbol{\theta_{t+1}} \leftarrow \boldsymbol{\theta_t}$.
      accepted $\leftarrow 0$.
    end if
  else
    $\boldsymbol{\theta_{t+1}} \leftarrow \boldsymbol{\theta_t}$.
    accepted $\leftarrow 0$.
  end if
  t++
end while

B5 Additional methods for simulation of canine patch-clamp experiment

Figure B2 shows the periods of the experiment which were taken for this study, and provides an impression of the beat-to-beat variability of one aspect of the APs — their duration. The full

[1]Something Spanish? Beat heard accompanying steps (9)

Algorithm 2 Adaptive Metropolis-Hastings MCMC continued from Algorithm 1.

loop
  $s \leftarrow t - 1000 \times$(number of parameters).
  $\gamma_s \leftarrow (s+1)^{-0.6}$.
  Given the current parameter state $\boldsymbol{\theta_t}$, sample $\boldsymbol{\theta^*} \sim \mathcal{N}(\cdot|\boldsymbol{\theta_t}, a_{s-1}\Sigma_{s-1})$.
  if $p(\boldsymbol{\theta^*}) \neq 0$ **then**
    Compute $\log(\alpha) = l(\boldsymbol{\theta^*}) - l(\boldsymbol{\theta_t})$.
    Sample $u \sim \mathcal{U}(0,1)$.
    if $u < \alpha$ **then**
      $\boldsymbol{\theta_{t+1}} \leftarrow \boldsymbol{\theta^*}$.
      accepted $\leftarrow 1$.
    else
      $\boldsymbol{\theta_{t+1}} \leftarrow \boldsymbol{\theta_t}$.
      accepted $\leftarrow 0$.
    end if
  else
    $\boldsymbol{\theta_{t+1}} \leftarrow \boldsymbol{\theta_t}$.
    accepted $\leftarrow 0$.
  end if
  $\Sigma_s \leftarrow (1-\gamma_s) \times \Sigma_{s-1} + \gamma_s \times (\boldsymbol{\theta_{t+1}} - \boldsymbol{\mu_{s-1}})^T(\boldsymbol{\theta_{t+1}} - \boldsymbol{\mu_{s-1}})$.
  $\boldsymbol{\mu_s} \leftarrow (1-\gamma_s) \times \boldsymbol{\mu_{s-1}} + \gamma_s \times \boldsymbol{\theta_{t+1}}$.
  $\log(a_s) \leftarrow \log(a_{s-1}) + \gamma_s \times (accepted - 0.25)$.
  t++
end loop

dataset is available to download as part of the code associated with this project.

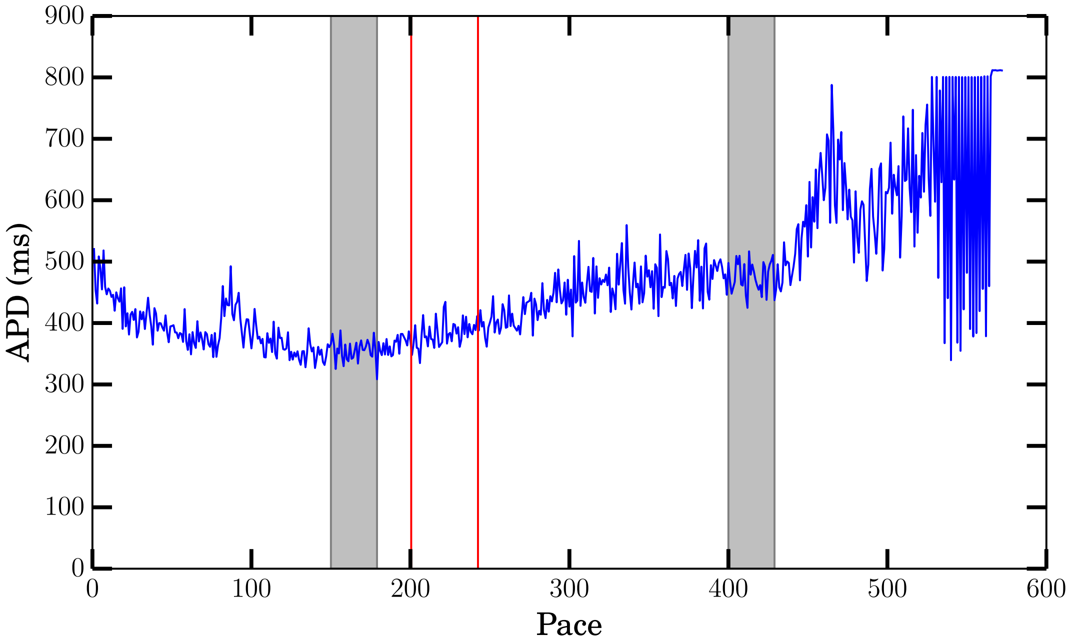

Figure B2: Summary of the whole patch clamp experiment. Action Potential Durations (APD_{90}) are shown for every pace in the experiment. The first grey shaded region, at paces #150–179, shows the training data, at control. The first red line shows when 10μM Moxifloxacin was added, the second when the bath KCl concentration was increased to 5.4mM (raising the extracellular potassium concentration). The second shaded region, at paces #400–429, highlights the validation data paces. The shaded regions were chosen to be representative of the patch-clamped cell having settled to roughly steady behaviour in control and altered conditions. After pace $\sim$ #450 the patch-clamp becomes unstable and action potentials lengthen until they are over 1 s long and we have 2 paces: 1 AP (at around pace #530, leading to an artefact in the plot by which the APD appears to be the full length of the trace, and then short on the next pace).

We adapted a method proposed by Dokos and Lovell (2004) to clamp the generated action potential to the experimental voltage trace during just that part of the upstroke attributable to the stimulus current.

The data clamp current takes the form

$$I_{\text{clamp}} = g_{\text{clamp}} \left(V - V_{\text{data}} \right). \tag{B.8}$$

Dokos and Lovell (2004) used the integral of I_{clamp} as an objective function to minimise. We simply use this method to ensure we are representing the effect of the stimulus current exactly, and then let the action potential evolve as normal, and continue to calculate the likelihood as before (see Section B2). To enable the CVODE solver to recover V_{data} at any time (it is taking adaptive time steps), linear interpolation is performed. We coded this feature into Chaste (`http://www.cs.ox.ac.uk/chaste`) so the upcoming release v3.4 will allow a data clamp to be applied to any CellML model. We switched on the data clamp by setting its conductance g_{clamp} to 300 mS/μF during the time period shown in Figure B3, and zero otherwise.

We inferred conductances from each AP independently according to the following procedure

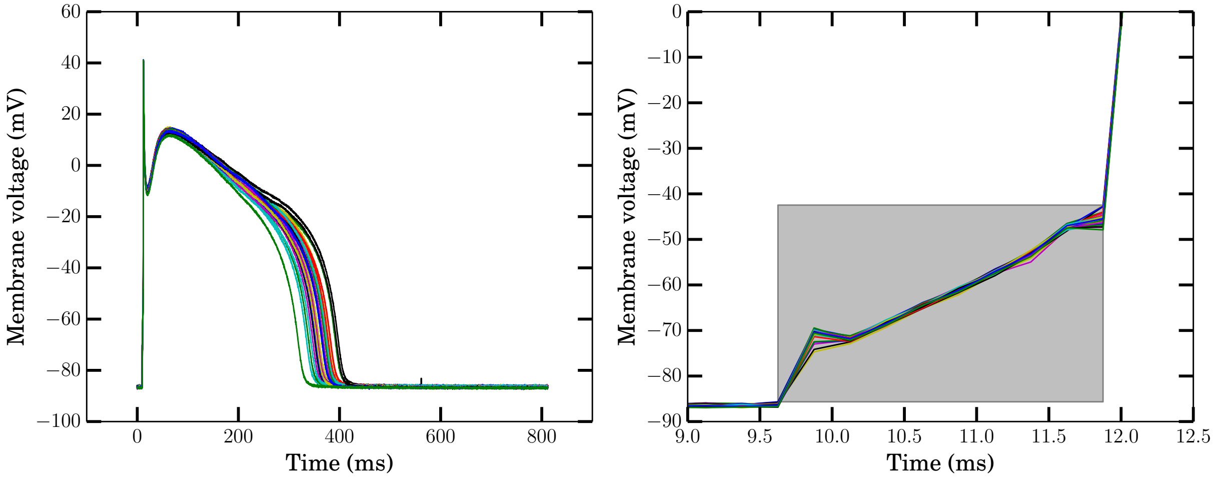

Figure B3: Left: Experimental action potential recordings of the 30 subsequent paces shown in Figure B2. Right: zoomed in view to show the effect of the experimentally-applied stimulus on membrane voltage. The grey box is overlaid to show the time in which we apply the data clamp current for each trace to ensure simulations follow the voltage time course throughout this time period.

- Set the intra- and extra-cellular potassium concentrations to the concentration of KCl in the experiment
- Set the initial voltage value to the initial voltage value in AP trace.
- Apply 'data clamp current' for a short time during the relevant part of the upstroke. We found this method was necessary to ensure that the main upstroke was provided by I_{Na} and not the applied stimulus current, particularly if assessing different models (not shown here).
- Turn off the data clamp allow the model to continue for a total of 1000 ms.
- Repeat this many times until the model reaches its steady state for its default parameters, defined by L_1 norm of difference in state variables after successive paces being less than 10^{-6}.
- Since the stimulus currents are highly consistent (Figure B3), we use the same steady state as initial conditions for fitting all traces.
- Perform CMA–ES runs followed by MCMC for each dog trace (#150–179), as described in the methods of the main text.

B6 Additional results for Section 2.2

Superimposed normalised histograms of inferred distributions for g_{K1}, g_{Kr} and g_{Ks} using the ten Tusscher et al. model under two protocols are given in Figure B4. Note that all inferred distributions in the $2[\mathrm{K}^+]_o$ protocol are shifted to the left, i.e. the inferred conductance values are generally smaller.

A summary of which conductances were successfully inferred from the ten Tusscher et al. (2004), O'Hara et al. (2011) and Davies et al. (2012) models is given in Table B1. All conductances were successfully inferred for the four simpler models.

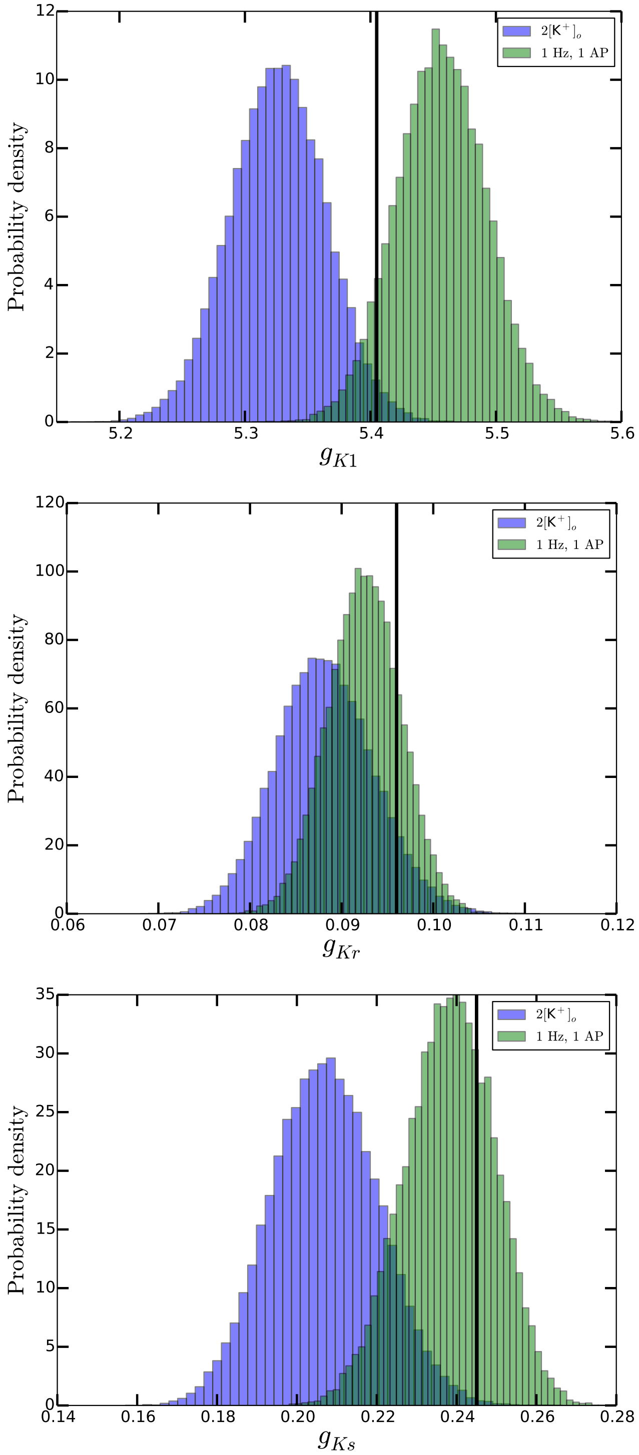

Figure B4: Superimposed normalised histograms for g_{K1}, g_{Kr} and g_{Ks} for two protocols using the ten Tusscher et al. model. The blue histograms are from the $2[K^+]_o$ protocol, the green are from the 1 Hz single AP protocol. The vertical black lines are the original values given in the model.

Parameter	Protocol										
	1 Hz 1 AP	$0.5[K^+]_o$ 1 AP	$2[K^+]_o$ 1 AP	1 & 2 Hz 3 APs	1 Hz for 2000 ms, $G_x = 0$ at 1000 ms: G_{CaL}	G_{K1}	G_{Kr}	G_{Ks}	G_{to}	1 Hz 10 s	1 Hz 20 s
**ten Tusscher et al. (2004)**											
G_{Na}	✓	✓	✓	✓	✓	✓	✓	✓	✓	✓	✓
G_{CaL}	✓	✓	✓	✓	✓	✓	✓	✓	✓	✓	✓
G_{K1}	✓	✓	✓	✓	✓	✓	✓	✓	✓	✓	✓
G_{Kr}	✓	✓	✗	✓	✓	✓	✓	✓	✓	✓	✓
G_{Ks}	✓	✓	✗	✓	✓	✓	✓	✓	✓	✓	✓
k_{NaCa}	✗	✗	✗	✓	✓	✓	✓	✗	✓	✓	✓
G_{to}	✓	✓	✓	✓	✓	✓	✓	✓	✓	✓	✓
G_{bCa}	✗	✗	✗	✗	✗	✗	✗	✗	✗	✓	✓
G_{bNa}	✗	✗	✗	✗	✗	✗	✗	✗	✗	✗	✓
G_{pCa}	✗	✗	✗	✓	✓	✓	✗	✗	✓	✓	✓
G_{pK}	✓	✓	✓	✓	✓	✓	✓	✓	✓	✓	✓
P_{NaK}	✗	✗	✗	✗	✗	✓	✗	✗	✗	✓	✓
**O'Hara et al. (2011)**											
G_{Na}	✓	✓	✓	✓	✓	✓	✓	✓	✓	✓	✓
G_{CaL}	✗	✓	✗	✓	✓	✓	✓	✓	✓	✓	✓
G_{bK}	✗	✗	✗	✓	✓	✓	✓	✓	✗	✓	✓
G_{K1}	✓	✓	✓	✓	✓	✓	✓	✓	✓	✓	✓
G_{Kr}	✓	✓	✗	✓	✓	✓	✓	✓	✓	✓	✓
G_{Ks}	✗	✗	✗	✓	✓	✓	✓	✓	✗	✓	✓
k_{NaCa}	✗	✗	✗	✓	✓	✓	✓	✓	✓	✓	✓
P_{NaK}	✗	✗	✗	✗	✓	✗	✓	✗	✗	✓	✓
G_{to}	✓	✓	✓	✓	✓	✓	✓	✓	✓	✓	✓
G_{bCa}	✗	✗	✗	✗	✗	✗	✗	✗	✗	✗	✓
G_{bNa}	✗	✗	✗	✗	✗	✗	✗	✗	✗	✗	✗
G_{pCa}	✗	✗	✗	✗	✗	✗	✗	✗	✗	✗	✗
G_{NaL}	✓	✓	✗	✓	✓	✓	✓	✓	✓	✓	✓
**Davies et al. (2012)**											
G_{Na}	✓	✓	✓	✓	✓	✓	✓	✓	✓	✓	✓
G_{CaL}	✓	✓	✓	✓	✓	✓	✓	✓	✓	✓	✓
G_{K1}	✓	✓	✓	✓	✓	✓	✓	✓	✓	✓	✓
G_{pK}	✗	✓	✗	✓	✓	✓	✓	✓	✓	✓	✓
G_{Ks}	✗	✗	✗	✗	✗	✗	✗	✓	✓	✓	✓
G_{Kr}	✗	✗	✗	✓	✓	✓	✓	✓	✓	✓	✓
G_{pCa}	✗	✗	✗	✗	✗	✗	✗	✗	✗	✗	✗
G_{bCa}	✗	✗	✗	✗	✗	✓	✓	✓	✗	✓	✓
k_{NaCa}	✗	✓	✓	✓	✓	✓	✓	✓	✓	✓	✓
P_{NaK}	✗	✗	✗	✓	✗	✓	✓	✓	✓	✓	✓
G_{to1}	✓	✓	✓	✓	✓	✓	✓	✓	✓	✓	✓
G_{to2}	✓	✗	✗	✓	✓	✓	✓	✓	✓	✓	✓
G_{bCl}	✗	✗	✗	✗	✗	✗	✗	✗	✗	✗	✗
G_{NaL}	✗	✗	✗	✓	✓	✓	✓	✓	✓	✓	✓

Table B1: Inference success for each conductance in the ten Tusscher et al. (2004), O'Hara et al. (2011) and Davies et al. (2012) models. The other four models had every parameter successfully inferred for every protocol.

B6.1 Prediction of beat-to-beat variability in a canine myocyte

Figure B5 shows the result of taking the maximum likelihood conductances that were inferred from each pace at control, and using these to predict changes to the action potential under the addition of 10 μM Moxifloxacin and 5.4 mM KCl rather than 4 mM, as described in Section 2.1.2. The model consistently predicted a lower resting potential, and a shorter APD than the experiment. This could be due to the experiment not having reached a steady-state response, the resting potential was still dropping at paces #400–429 (not shown, but full dataset is available to download). The original model parametrisation behaves the same way, so whatever the cause, this is not purely a product of our re-parametrisation procedure.

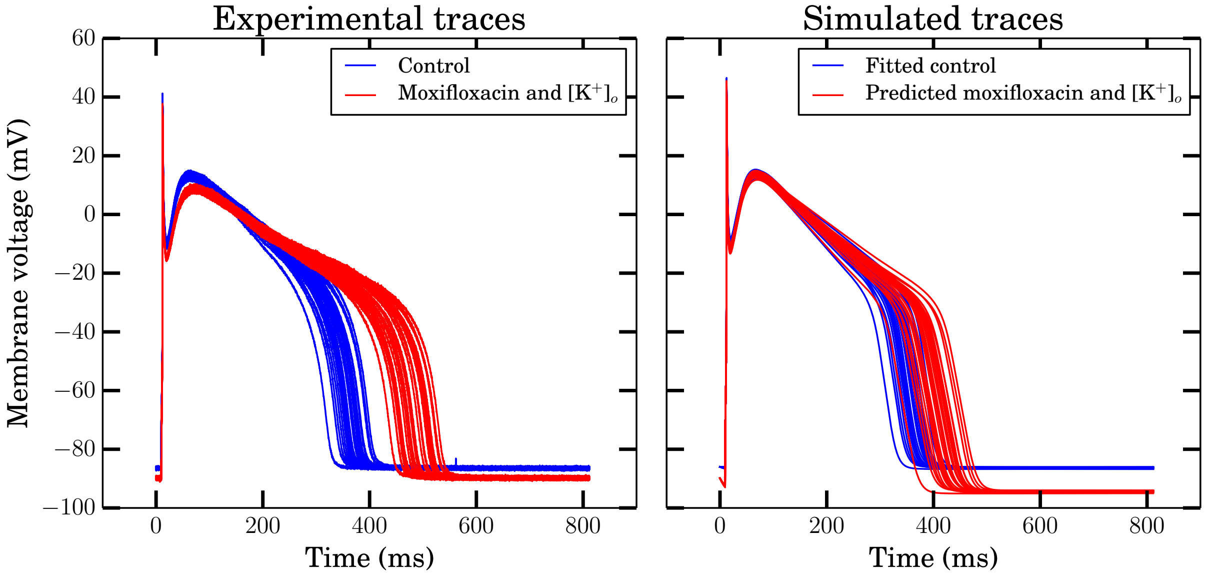

Figure B5: Left: blue — experimental APs #150–179, and red — experimental APs #400–429. Right: blue — simulated APs using maximum likelihood conductances inferred from experimental paces #150–179, and red — predicted APs using the same parameters under Moxifloxacin and raised extracellular potassium concentration.

In Figure B6 we plot the dispersion of APDs that are present in the data shown in Figure B5. There is a good correspondence in predicted beat-to-beat variability, with an increase relative to the control situation. The slight underestimate of variability in the simulation predictions is consistent with the observation that longer APDs lend themselves to higher variability (Heijman et al., 2013), and so we would expect that a model which reproduced the baseline response more closely may also improve the beat-to-beat variability predictions.

The fact that the model predictions are not very good for the absolute APD and resting potential indicates that Structural Uncertainty may be playing a large role. This may be caused by differences between the experimental preparation and its approximation in our simulations, rather than simply the model being inadequate/untested for simulating altered extracellular potassium and block of ion channels.

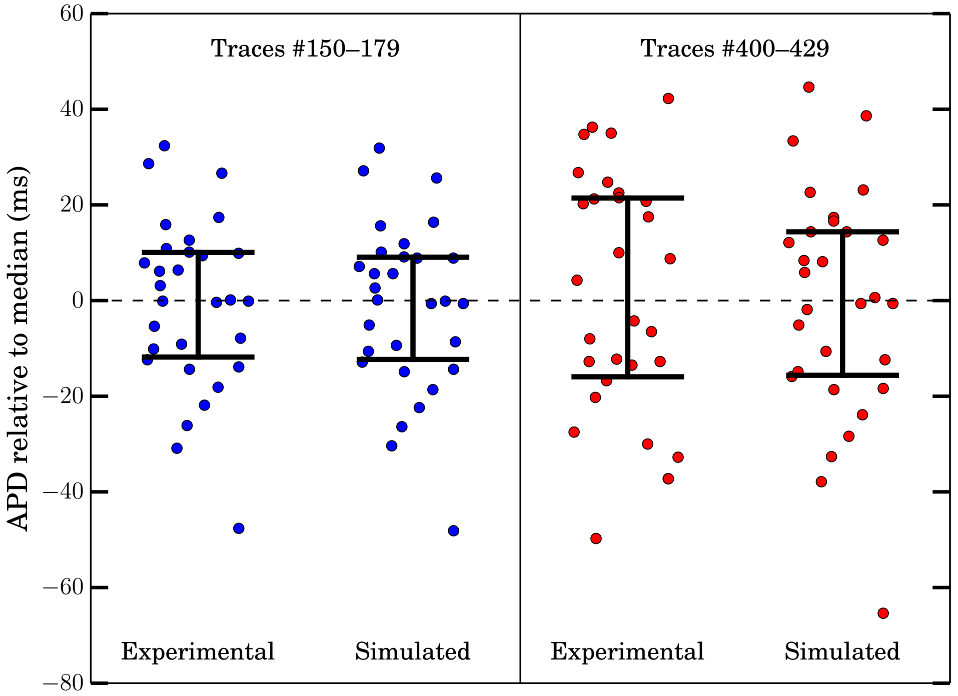

Figure B6: Dispersion of APD in the datasets shown in Figure B5. Left: in the training dataset, from traces #150–179. Right: in the prediction dataset, traces #400–429. Quartiles overlaid.

C Case Study 2: introduction to GP emulators

A Gaussian Process is a distribution over functions, that generalises classical regression (Rasmussen and Williams, 2006). A GP is parametrized by two functions: a mean function and a covariance function. The GP with mean function $m(\mathbf{x})$ and covariance function $c(x, x')$ is the unique distribution on function f such that given any number of fixed test inputs $\mathbf{x_1}, \ldots, \mathbf{x_n}$, the values of f at $\mathbf{x_1}, \ldots, \mathbf{x_n}$ are jointly Gaussian, with mean $m(\mathbf{x_1}), \ldots, m(\mathbf{x_n})$, and with covariance matrix $c(\mathbf{x_i}, \mathbf{x_j})$. Thus, m describes a trend, while c encodes how much the value of f at different inputs should be correlated. GPs have been widely used in statistics, as they enjoy many desirable computational properties. In particular, conditioning a GP on training points, where on has evaluated the target function, still yields a GP posterior distribution on f, the mean and covariance functions of which can be analytically computed.

In this supporting information, we provide a simple example of emulation, and then include details of the mathematics that underpin the GP emulator work described in Case Study 2. A much more in-depth coverage of Gaussian process (GP) emulators is given in the MUCM webpages and the toolkit that has been developed there `http://mucm.aston.ac.uk/MUCM/MUCMToolkit/index.php`. Our aim here is to describe the pathway through this material that was followed for the present study.

C1 Simple example

Figure C7 illustrates the results of GP emulation of a simple hypothetical computational model. For this example, the computational model is assumed to be dependent on one input parameter,

and provides one output parameter of interest. Suppose this computational model is expensive to run, but that the model has been run at 6 values of model input parameter, $(0, 0.2, 0.4, 0.6, 0.8, 1)$. These values, and the corresponding model outputs, are the design data for the emulator. The emulator is constructed from the design data, and the result includes an expectation function (solid line in figure), which is the prediction of what the model output is at other inputs, and can be used as a surrogate for the original computational model. The emulator also provides a variance function (represented by the shaded region), which represents uncertainty of the emulator output as a function of model input values. Therefore, the variance is zero at design points (where there is no uncertainty since the model was run at these points), and increases away from design points.

In general, a *meta-model* is defined as a surrogate for a model (for example, a simple polynomial interpolant through the data points in Figure C7), and an emulator is defined as a meta-model that also provides uncertainty in the prediction.

Once constructed, an emulator can be tested (validated) by running the model again (e.g. at input = 0.5 say) and comparing the emulator prediction with the model output, given the emulator uncertainty. If the model result at this new input is several standard deviations away from the expected value, this suggests the emulator requires improvement (by using more design data, say).

Now suppose we have some real data on the input parameter, and can generate a probability density function for it. This is represented in the figure with the distribution illustrated on the input axis; here we have assumed the input was observed to have mean value 0.8 and standard deviation 0.04. (This is the uncertainty characterisation stage). Then, the uncertainty propagation stage involves computing the corresponding distribution of the model output, which is also illustrated in the figure. This could be performed using Monte Carlo sampling and repeatedly running the original model (which could be infeasible if the model is very expensive to run), or alternatively using Monte Carlo sampling and repeatedly evaluating the emulator predictions. However, if the input is discovered or assumed to be Normally distributed, there is an analytic description of the mean and variance of the output, which can be computed directly from emulator properties. (The emulator's uncertainty regarding the model output is also accounted for in this analytic description). Therefore, uncertainty propagation can be performed without the need for additional sampling. Hence, GP emulators can be especially useful even for computational models that are not very expensive to solve.

C2 Emulator implementation

The GP emulators used in this study were constructed using the approach described in the MUCM web pages, and full mathematical details are given in the supporting information to (Chang et al., 2015).

Briefly, each TP06 emulator was described by a Gaussian process (GP),

$$\mathcal{GP}(\mathbf{x}) = h(\mathbf{x})^T\beta + \sigma^2 c(\mathbf{x}, \mathbf{x}'). \tag{C.9}$$

which was composed of a linear mean function $m(\mathbf{x}) = h(\mathbf{x})^T\beta$, where $h(\mathbf{x}) = (1, \mathbf{x})^T$ such that

$$h(\mathbf{x})^T\beta = \beta_0 + \beta_1 x_1 + ... + \beta_P x_P, \tag{C.10}$$

and a Gaussian covariance function $\sigma^2 c(\mathbf{x}, \mathbf{x}')$, with

$$c(\mathbf{x}_1, \mathbf{x}_2) = \exp\left[-\sum_{p=1}^{P}\left\{\frac{(x_{p,1} - x_{p,2})}{\delta_p}\right\}^2\right]. \tag{C.11}$$

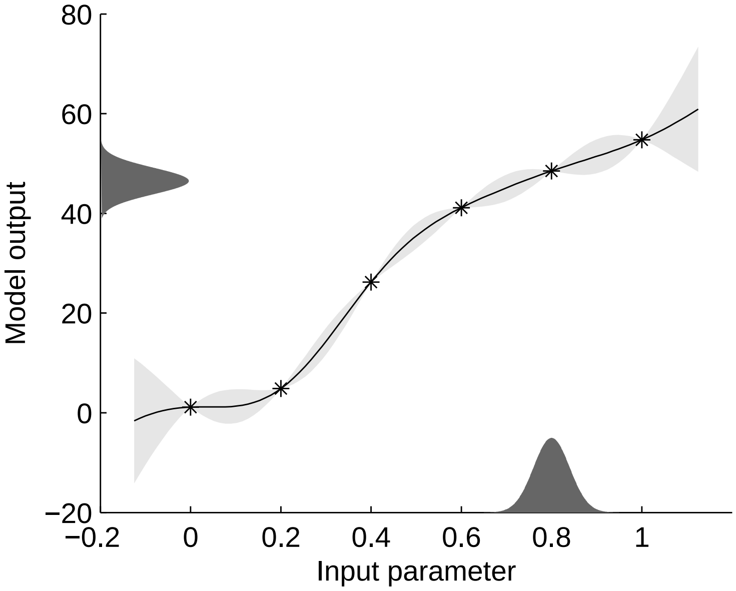

Figure C7: Emulation of a model that takes in a single input. Stars: inputs and corresponding outputs at which the original model was run. Line: expectation function of the emulator, i.e. the predicted value of the model at other inputs. Light shaded area: a representation of the uncertainty of the prediction by the emulator—one standard deviation above and below the predicted value. Dark shaded area: illustration of an example input distribution that might be observed from data (uncertainty characterisation stage), and illustration of the corresponding output distribution (uncertainty propagation stage). This figure was created by recreating the MUCM example `http://mucm.aston.ac.uk/MUCM/MUCMToolkit/index.php?page=ExamCoreGP1Dim.html`, although the absolute values were altered.

In these equations $\mathbf{x} = (x_1, x_2, \ ... \ , x_P)$ are P model inputs, β and δ are vectors of length P, and σ^2 is a scalar. The emulator is specified by the hyperparameters β, δ, and σ^2. These hyperparameters were obtained by fitting the emulator to the design data, assuming a flat prior on δ of 1.0, and then maximising the posterior likelihood given the design data as detailed in (Chang et al., 2015).

C3 Emulator validation

The emulators were validated by generating an additional set of test data, comprising inputs and outputs from 10 model runs. For each set of inputs in the test data, the outputs obtained from the emulator were compared with the outputs obtained from the AP model for the same inputs, and the differences were compared using the Mahanalobis distance (Bastos and O'Hagan, 2009; Chang et al., 2015)

The Mahalanobis distance for the complete set of test data was a measure of overall agreement between the predicted and test data. The results are summarised in Table C2, where the second column shows the reference distribution for the Mahanalobis distance. For design data with 50 sets of input and output data, the Mahalanobis distance for each emulator was within one standard deviation of the mean of the reference distribution, so these emulators were judged to be a good trade off between emulator fit and the number of model runs required to generate the design data, and were used for all the results described in the main text.

Table C2: Mahalanobis distance for each of the six TP06 emulators: fitted with $n = 25$, 50 and 100 design points.

Design data	Mean (SD)	Max dV_m/dt	Max V_m	Dome V_m	APD_{90}	Rest V_m	APD_{50}
$n = 25$	10 (12.87)	18.2897	13.0487	10.1815	15.6885	14.2972	18.2897
$n = 50$	10 (5.24)	10.7534	5.8113	5.488	7.4894	8.9025	10.3506
$n = 100$	10 (7.12)	10.5641	11.1173	17.5544	10.0706	10.8453	9.6464

C4 Emulator Implementation

All of the code for this study was implemented in Matlab, using expressions detailed in the MUCM toolkit (`http://mucm.aston.ac.uk/MUCM/MUCMToolkit/`). The code was tested against the numerical examples provided in the toolkit.

Further details of the methods used to calculate the mean effects and sensitivity indices are given in (Chang et al., 2015).

References

Bastos, L. S., O'Hagan, A., Nov. 2009. Diagnostics for Gaussian Process Emulators. Technometrics 51 (4), 425–438.

Chang, E., Strong, M., Clayton, R., 2015. Bayesian sensitivity analysis of a cardiac cell model using a Gaussian process emulator. PLoS ONE 10 (6), e0130252.

Cooper, J., Spiteri, R. J., Mirams, G. R., Jan. 2014. Cellular cardiac electrophysiology modeling with Chaste and CellML. Frontiers in Physiology 5, 511.

Cordeiro, J., Mazza, M., Goodrow, R., Ulahannan, N., Antzelevitch, C., Di Diego, J., 2008. Functionally distinct sodium channels in ventricular epicardial and endocardial cells contribute to a greater sensitivity of the epicardium to electrical depression. American Journal of Physiology-Heart and Circulatory Physiology 295 (1), H154–H162.

Davies, M., Mistry, H., Hussein, L., Pollard, C., Valentin, J.-P., Swinton, J., Abi-Gerges, N., 2012. An in silico canine cardiac midmyocardial action potential duration model as a tool for early drug safety assessment. American Journal of Physiology - Heart and Circulatory Physiology 302 (7), H1466–H1480.

Dokos, S., Lovell, N. H., 2004. Parameter estimation in cardiac ionic models. Progress in biophysics and molecular biology 85 (2-3), 407–31.

Hansen, N., 2006. The cma evolution strategy: a comparing review. In: Towards a new evolutionary computation. Springer, pp. 75–102.

Heijman, J., Zaza, A., Johnson, D. M., Rudy, Y., Peeters, R. L. M., Volders, P. G. A., Westra, R. L., Aug. 2013. Determinants of Beat-to-Beat Variability of Repolarization Duration in the Canine Ventricular Myocyte: A Computational Analysis. PLoS Computational Biology 9 (8), e1003202.

Hunter, J. D., 2007. Matplotlib: A 2d graphics environment. Computing in science and engineering 9 (3), 90–95.

Lloyd, C., Lawson, J., Hunter, P., Nielsen, P., 2008. The CellML model repository. Bioinformatics 24 (18), 2122–2123.

Mirams, G., Arthurs, C., Bernabeu, M., Bordas, R., Cooper, J., Corrias, A., Davit, Y., Dunn, S.-J., Fletcher, A., Harvey, D., Marsh, M., Osborne, J., Pathmanathan, P., Pitt-Francis, J., Southern, J., Zemzemi, N., Gavaghan, D., 2013. Chaste: an open source C++ library for computational physiology and biology. PLOS Computational Biology 9 (3), e1002970.

Murphy, L., Renodin, D., Antzelevitch, C., Di Diego, J., Cordeiro, J., 2011. Extracellular proton depression of peak and late Na+ current in the canine left ventricle. American Journal of Physiology—Heart and Circulatory Physiology 301 (3), H936–H944.

O'Hara, T., Virág, L., Varró, A., Rudy, Y., 2011. Simulation of the undiseased human cardiac ventricular action potential: model formulation and experimental validation. PLoS computational biology 7 (5), e1002061.

Pathmanathan, P., Shotwell, M. S., Gavaghan, D. J., Cordeiro, J. M., Gray, R. A., 2015. Uncertainty quantification of fast sodium current steady-state inactivation for multi-scale models of cardiac electrophysiology. Progress in Biophysics and Molecular Biology 117 (1), 4–18.

Rasmussen, C. E., Williams, C. K. I., 2006. Gaussian Processes for Machine Learning. MIT Press.

ten Tusscher, K., Noble, D., Noble, P. J., Panfilov, A. V., 2004. A model for human ventricular tissue. Am. J. Physiol. Heart Circ. Physiol. 286 (4), 1573–1589.

Van Der Walt, S., Colbert, S. C., Varoquaux, G., 2011. The numpy array: a structure for efficient numerical computation. Computing in Science & Engineering 13 (2), 22–30.
